# SQOR regulates H_2_S detoxification and lipid metabolism to maintain glioblastoma metabolic fitness

**DOI:** 10.21203/rs.3.rs-11077727/v1

**Published:** 2026-09-28

**Authors:** Andras K. Ponti, Taylor Gatesman, Sarah Hatem, Jordyn Smith, Ishana Senthil, Arjun Akula, Lilly Stanley, Matthew Godwin, Kewal Asosingh, Isaac Ampong, Belinda Willard, Naseer Sangwan, Andrew Dhawan, Daniel J. Silver, Sameer Agnihotri, Justin D. Lathia, Christopher Hine

**Affiliations:** 1 Department of Genomic Sciences and Systems Biology, Cleveland Clinic Research, Cleveland, OH, USA; 2 Department of Cancer Sciences, Cleveland Clinic Research, Cleveland, OH, USA; 3 Department of Molecular Medicine, Cleveland Clinic Lerner College of Medicine, Case Western Reserve University, Cleveland, OH, USA; 4 Case Comprehensive Cancer Center, Case Western Reserve University, Cleveland, OH, USA; 5 Department of Pathology, University of Pittsburgh, Pittsburgh, PA, USA; 6 Department of Neurological Surgery, University of Pittsburgh, Pittsburgh, PA, USA; 7 Department of Heart, Blood, and Kidney Research, Cleveland Clinic Research, Cleveland, OH, USA; 8 Department of Inflammation and Immunity, Cleveland Clinic Research, Cleveland, OH, USA; 9 Shared Laboratory Resource, Cleveland Clinic Research, Cleveland, OH, USA; 10 Department of Microbial Sciences in Health, Cleveland Clinic Research, Cleveland, OH, USA; 11 Rose Ella Burkhardt Brain Tumor & Neuro-Oncology Center, Cleveland Clinic, Cleveland, OH, USA; 12 Department of Pathology, Cleveland Clinic Lerner College of Medicine, Case Western Reserve University, Cleveland, OH, USA; 13 Pediatric Neuro-Oncology Program, UPMC Children’s Hospital of Pittsburgh, Pittsburgh, PA, USA; 14 Department of Clinical Medicine, Faculty of Health and Medical Sciences, University of Copenhagen, Copenhagen, Denmark; 15 These authors contributed equally; 16 Lead contacts

**Keywords:** Glioblastoma, GBM, Hydrogen sulfide, H_2_S, Sulfide quinone oxidoreductase, SQOR, SQR, SQRDL, Cystathionine gamma lyase, CGL, CTH, CSE, Transsulfuration, Sulfide oxidation, TCA cycle, Krebs Cycle, Mitochondria, Lipid metabolism, Lipid droplets, Cancer metabolism

## Abstract

Glioblastoma (GBM) adapts to metabolic stress through mitochondrial pathways that are not fully defined. Here, we show sulfide quinone oxidoreductase (SQOR) is upregulated in human GBM and functions as a mitochondrial checkpoint linking hydrogen sulfide (H_2_S) clearance to electron transport chain activity. In patient-derived GBM cells and immunocompetent syngeneic tumors, SQOR channels H_2_S-derived electrons into the ubiquinone pool to sustain oxidative phosphorylation during stress. Genetic or pharmacologic SQOR inhibition causes intracellular H_2_S accumulation, collapse of mitochondrial respiration, tricarboxylic acid cycle dysfunction, and compensatory glycolytic engagement. SQOR loss also redirects carbon into lipid synthesis and storage while impairing lipid mobilization, thereby limiting metabolic flexibility and stem-like cell maintenance. Increasing endogenous H_2_S production further exacerbates the bioenergetic defects caused by SQOR blockade. Functionally, genetic or pharmacologic SQOR inhibition suppresses tumor growth and prolongs survival *in vivo*. Together, these data identify SQOR-dependent sulfide oxidation as a mitochondrial liability that supports GBM metabolic plasticity.

## Expanded Summary

Glioblastoma (GBM) thrives in conditions of metabolic stress by engaging adaptive pathways that remain incompletely defined. Here, we uncover mitochondrial sulfide oxidation as an unappreciated organizing principle of GBM metabolic fitness and identify sulfide quinone oxidoreductase (SQOR) as a central node in this program. We show elevation of SQOR in patient tumors and demonstrate how this enzyme serves two roles in the tumor microenvironment, first as a detoxification enzyme, second as a metabolic shunt driving tumor-suppressive metabolite hydrogen sulfide (H_2_S) into mitochondrial electron transport, thereby sustaining oxidative metabolism under stress. Using patient-derived GBM cells and immunocompetent syngeneic GBM models, we find that genetic or pharmacologic SQOR inhibition unleashes intracellular H_2_S accumulation, precipitating a broad bioenergetic crisis marked by loss of cellular fitness, self-renewal, impaired tumor growth, and prolonged survival in tumor-bearing mice. Integrated metabolomic, transcriptional, and functional analyses reveal that SQOR loss triggers mitochondrial failure, disrupts tricarboxylic acid cycle homeostasis, and enforces a compensatory shift toward glycolysis. Concomitantly, SQOR blockade rewires lipid metabolism, promoting lipid synthesis and storage while uncoupling these reserves from productive use during energetic stress and diminishing the cancer stem cell phenotype. Increasing endogenous H_2_S production through cystathionine γ-lyase overexpression further intensifies this collapse, exposing a fundamental dependence of GBM cells on sulfide disposal. Together, our findings redefine sulfide oxidation as a tumor-supportive mitochondrial circuit and establish SQOR as a previously unrecognized metabolic checkpoint that integrates H_2_S detoxification, electron flow, and lipid homeostasis in adaptive GBM cells. These results reveal a conceptual framework in which sulfide metabolism is co-opted to preserve tumor resilience and propose SQOR as a therapeutic entry point for disabling metabolic plasticity in GBM.

## Graphical Abstract

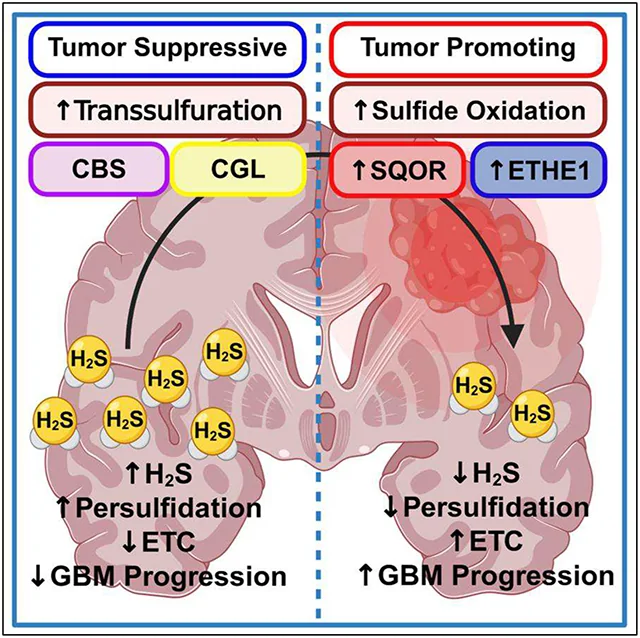

## Introduction

Glioblastoma (GBM) is one of the most lethal human cancers, defined by diffuse invasion, profound intratumoral heterogeneity, and near-universal recurrence despite maximal therapy. Combined, multimodal therapy under the Stupp protocol, involving maximal safe surgical resection followed by radiotherapy and temozolomide chemotherapy extends median survival from 6–8 months without treatment, to 12–15 months^1,2^. The persistence of poor outcomes reflects not only genomic heterogeneity and invasive growth but also profound tumor-intrinsic metabolic plasticity that enables glioblastoma cells to adapt to spatially heterogeneous environments, fluctuating oxygen, variable nutrient abundance, and altered redox homeostasis within the brain^3,4^.

Metabolic reprogramming in GBM extends well beyond enhanced glycolysis to encompass dynamic modulation of mitochondrial respiration^5–7^, lipid metabolism^8–11^, amino acid metabolism^12^, and antioxidant systems^13^. Tumor cells, particularly in GBM, maintain a high degree of metabolic flexibility, enabling adequate ATP production while simultaneously preserving nucleotide, amino acid, and lipid building blocks for synthesis of DNA, proteins, and lipid rich membranes. However, during periods of low nutrient availability, cancerous cells can meet their energetic demands by switching from glycolysis to utilization of lipids and amino acids. These same mechanisms enable malignant cells to develop resistance to radiation and chemotherapy, while finely tuning redox buffering networks to avoid oxidative catastrophe^14,15^. Among the metabolic axes increasingly recognized as critical in this context is sulfide metabolism^16^, which integrates methionine and cysteine availability, glutathione synthesis, hydrogen sulfide (H_2_S) production, and reactive sulfur species signaling^17–19^. Notably, sulfide metabolism is uniquely positioned at the intersection of bioenergetics and redox control, which are two core determinants of GBM cell survival^20,21^.

The transsulfuration pathway (TSP) is a major arm of sulfur amino acid metabolism, converting homocysteine to cysteine through the sequential actions of cystathionine β-synthase (CBS) and cystathionine γ-lyase (CGL, also known as CSE or CTH). This pathway supports the generation of key downstream metabolites, including glutathione, while hydrogen sulfide (H_2_S) is produced through sulfur metabolic reactions mediated by CBS, CGL, and 3-mercaptopyruvate sulfurtransferase (MPST, also known as 3-MST).^22^. Among these, CGL has emerged as a particularly relevant regulator of tumor-intrinsic sulfide production and bioavailability^23,24^. Canonically, CGL catalyzes the conversion of cystathionine to cysteine, α-ketobutyrate, and ammonia, thereby contributing to intracellular cysteine pools that support glutathione synthesis and redox balance^25–27^. CGL can also generate H_2_S directly from cysteine or homocysteine, linking TSP activity to sulfide signaling. H_2_S, once regarded solely as a toxic gas, is now understood to function as an endogenous gasotransmitter with context and concentration-dependent effects on mitochondrial activity and cell fate^28,29^.

In GBM, cysteine and sulfide metabolism emerge as a paradoxical but potentially central axis of tumor biology. Increased cysteine availability and cystathionine γ-lyase (CGL) activity have been associated with enhanced antioxidant capacity and pro-tumorigenic behavior^30–34^, largely through glutathione synthesis that enables malignant cells to withstand the high burden of reactive oxygen species (ROS) and lipid peroxides intrinsic to the transformed state, thereby promoting chemoresistance and evasion of ferroptotic death^35–37^. Yet this framework is incomplete, as elevated sulfide metabolism, CGL activity, and H_2_S have also been shown to exert striking tumor-suppressive effects through proposed cell-intrinsic mechanisms^34,38–43^. This apparent contradiction suggests that the critical output of sulfide metabolism may not be antioxidant buffering alone, but the selective use and/or propagation of sulfide-based signaling through protein persulfidation (aka S-sulfhydration), a reversible post-translational modification that converts cysteine thiols to persulfide (-SSH) groups^44–46^. By reshaping protein conformation, activity, and susceptibility to irreversible overoxidation on cysteine residues, persulfidation is increasingly recognized as a mechanism capable of rewiring glycolytic flux^47^, mitochondrial electron transport^48^, transcriptional programs^49^, and cell death pathways^50,51^: all processes that collectively define malignant fitness. These observations raise the possibility that the balance between sulfide’s role in tumor promotion and tumor suppression in GBM are conditional, and dictated not simply by sulfide metabolite abundance, but how sulfide is fixed into the proteome or used for downstream metabolic processes in both a cell-intrinsic and cell-extrinsic manner has yet to be determined.

Despite the clear oncogenic advantages of maintaining an elevated cysteine pool, catabolism of sulfur containing amino acids and generation of H_2_S presents a severe metabolic liability for gliomas. This paradox suggests that the biological consequence of sulfide metabolism in GBM may not be determined solely by the rate of H_2_S production, but by the relative concentrations determined by the balance between H_2_S generation and disposal^52,53^. The ability to counteract the biological impact of H_2_S and persulfidation is tightly controlled by sulfide oxidation pathways^54^, particularly through the mitochondrial enzyme sulfide quinone oxidoreductase (SQOR, SQR, or SQRDL)^55^. SQOR catalyzes the first and rate-limiting step of sulfide oxidation, transferring electrons from H_2_S to coenzyme Q within the electron transport chain^56,57^. Subsequent reactions are catalyzed by downstream enzymes, including persulfide dioxygenase (ETHE1 or PDO), rhodanese (TST), and sulfite oxidase (SUOX) required to further process sulfur intermediates for complete detoxification^58^. This series of reactions initiated by SQOR not only prevents mitochondrial-localized and/or systemic toxic sulfide accumulation^58–60^ but also regulates the steady-state levels of reactive sulfur species that drive persulfidation^46^ in a process that simultaneously has the benefit of supporting ATP production through the electron transport chain (ETC)^61^. In GBM cells, the balance between H_2_S production and SQOR-mediated sulfide clearance may determine the conditions in which sulfide functions as a bioenergetic substrate, a redox modulator, or a cytotoxic stressor.

Notably, although GBM extensively remodels its surrounding microenvironment^62–64^, current evidence linking sulfide metabolism to GBM has focused largely on tumor cell-intrinsic processes. Changes in CGL expression, cysteine utilization, SQOR activity, and global protein persulfidation suggest that sulfide metabolic pathways can directly reprogram mitochondrial respiration, redox homeostasis, and metabolic enzyme function within malignant cells. These observations raise the possibility that sulfide metabolism serves as a previously underappreciated mechanism by which GBM sustains metabolic adaptability under the nutrient, oxidative, and hypoxic stresses imposed by the brain tumor niche^65^. However, whether sulfide oxidation constitutes an actionable metabolic defense pathway in GBM, and whether this axis can be leveraged therapeutically, has remained unknown.

Here, we identify sulfide oxidation as a previously unrecognized and translationally relevant metabolic vulnerability in GBM. We show that cysteine metabolism is elevated in tumor-bearing brains and is coupled to induction of the sulfide oxidation machinery, most prominently SQOR. Using complementary genetic and pharmacologic perturbations in murine and human glioma models, we demonstrate that SQOR is a conditional dependency that shields GBM cells from both endogenous and microenvironment-derived H_2_S. SQOR loss drives intracellular sulfide accumulation, suppresses mitochondrial respiration, accumulates TCA-cycle intermediates, increases reliance on glycolysis, and redirects lipid metabolism from fatty acid oxidation toward known tumor-suppressive lipid synthesis and storage^66^. Together, these findings establish SQOR not merely as a detoxification enzyme, but as a central regulator of mitochondrial function and metabolic plasticity in GBM, and reveals sulfide oxidation as a targetable pathway with clear therapeutic potential in this otherwise intractable disease.

## Results

### Glioblastoma enhances cysteine metabolism and upregulates sulfide oxidation to buffer tumor-suppressive hydrogen sulfide

Metabolic flexibility is key to GBM initiation and progression^12,66^. To identify the GBM-associated metabolic changes, we performed untargeted metabolomics on the tumor-bearing and contralateral hemispheres of C57BL/6J mice implanted with the KR158 murine glioma model and compared to age- and sex-matched mock-implanted non-tumor bearing mice. Consistent in both tumor-bearing and non-tumor-bearing hemispheres was an increase in cysteine metabolism as a top three metabolic pathway enrichment ratio (Figures 1A and S1A). We next sought to test the impact of perturbations in cell extrinsic cysteine metabolism on tumor progression *in vivo* by utilizing a pair of transgenic models in which CGL is either constitutively knocked out (CGL KO) or present/overexpressed (CGL TG)^44,67–70^. Eight-week-old, male and female CGL KO and CGL TG mice were intracranially implanted with the KR158 murine glioma model that had no tumor-intrinsic manipulation of its sulfide metabolic pathways and monitored for disease progression and survival. In the absence of systemic CGL, a robust survival extension was surprisingly observed (Figure 1B). H_2_S production capacity was increased in both the tumor and non-tumor bearing hemispheres of CGL TG mice relative to the CGL KO mice (Figure 1C). These findings were of great interest as prior studies had shown that CGL and/or its metabolite H_2_S (Figure S1B) act as tumor suppressors in the context of glioblastoma^38,39,41,71^.

To elucidate this paradoxical finding and to expand upon the tumor-supportive versus tumor-suppressive roles of cysteine metabolism and H_2_S, we explored the role of the sulfide oxidation pathway (Figure 1D) as a potential mechanism by which GBM eliminates and/or co-opts H_2_S^57^. Western blot analysis of sulfide oxidation enzymes identified an increase in sulfide quinone oxidoreductase (SQOR) and persulfide dioxygenase (ETHE1) in the tumors of male and female CGL TG mice when compared to tumors from CGL KO mice, while rhodanese (TST) and sulfite oxidase (SUOX) were unchanged (Figures 1E and S1C). Additionally, we found that SQOR levels were greater in the tumor bearing hemisphere of C57BL/6J mice than was observed in the contralateral brain hemisphere (Figure 1F). Analysis of sulfide oxidation and production enzyme levels and H_2_S production capacity across a panel of human glioblastoma derived cell models indicated heterogenous expression and production patterns, potentially indicating differential and/or cell-line specific baseline requirements for H_2_S and its detoxification when in culture (Figures S1D–S1F). Screening of murine glioma models indicated similar variability in expression and activity of sulfide oxidation and transsulfuration enzymes (Figures S1G–S1I). However, when human and murine glioma cells are exposed to exogenous H_2_S administered with the slow-release donor molecule, GYY4137, there is both a dose and time dependent increase in sulfide oxidation levels (Figures 1G–1I). As the sulfide oxidation pathway appears to play a vital role in shielding malignant GBM cells from the tumor suppressive effects of H_2_S, we assessed TCGA and GTex data and found that SQOR and ETHE1 expression increases with tumor grade and beyond what is observed in non-tumor tissue samples (Figure 1J–1M). However, TST and SUOX expression were not associated with tumor grades or significantly elevated in tumor versus non-tumor samples (Figures S1J–S1M). Overall, these findings implicate increased sulfide oxidation function in GBM via SQOR and ETHE1 as a mechanism used to eliminate tumor suppressive H_2_S.

### SQOR deficiency suppresses GBM growth *in vitro* and *in vivo*

To investigate the functional role the sulfide oxidation pathway plays a role in GBM, we first generated SQOR knockdown murine glioma cells in the well-studied KR158^72^ and NPD^73,74^ GBM models for intracranial implantation (Figure 2A). Validation of SQOR knockdown in murine glioma cells using three independent shRNA constructs was confirmed by western blotting (Figure 1B–1C). SQOR knockdown enhanced H_2_S production capacity as determined by lead acetate assay (Figure S2A) and resulted in a robust anti-proliferative effect in murine glioma cells (Figures S2B). Decreased proliferation of murine glioma cells following SQOR knock down in turn lead to a robust survival extension *in vivo* in both syngeneic KR158 and NPD intracranially implanted mice (Figures 2D and 2E). The effect of SQOR knockdown on diminished proliferation was also observed in human HW1 GBM cells (Figure 2F), with validation of knockdown confirmed in Figure 2G, and attenuated colony formation used as a second measure of decreased GBM cell fitness (Figure 2H).

Genetic perturbation of SQOR significantly affected glioma fitness and survival in preclinical models, prompting us to evaluate the effects of pharmacologic SQOR inhibition *in vitro* and *in vivo* using FC9402 and HTS07545^75,76^. Consistent with the effect of knockdown, inhibition of SQOR activity with both FC9402 and HTS07545 inhibited colony formation in murine KR158 glioma cells (Figure S2C). Confirmation of SQOR inhibition was performed by flow cytometry using SF7-AM, a cell trappable, fluorescent H_2_S probe. Both SQOR inhibitors demonstrated potent activity leading to accumulation of intracellular H_2_S (Figures S2D and S2E). However, the kinetics of H_2_S accumulation were different between FC9402 and HTS07545, with levels rising more rapidly in cells treated with the latter.

We next evaluated the effect of pharmacological SQOR inhibition on *in vivo* survival in GBM models as our *in vitro* work indicated blocking SQOR decreased cell fitness. Survival studies testing the effect of pharmacological SQOR inhibition were performed in C57BL/6J mice implanted with either KR158 or NPD murine glioma cells. Seven days post-implantation, mice were administered vehicle control or FC9402 (0.5mg/kg) treatments via intraperitoneal (IP) injection three times weekly until endpoint (Figure 2I). Measurement of circulating H_2_S was performed with the fluorescent SF7-AM probe in plasma collected at endpoint. Inhibition of SQOR was confirmed to result in increased circulating H_2_S (Figure 2J). Importantly, blocking SQOR activity with the pharmacological inhibitor provided robust extension in survival in both KR158 and NPD bearing mice (Figures 2K and 2L) that was in line with our genetic SQOR knockdown results (Figures 2D and 2E). Consistent with our *in vitro* and *in vivo* findings using murine models, inhibition of SQOR in human GBM (HW1 and SB2b) cells with both FC9402 and HTS07545 resulted in decreased proliferation (Figures 2M and S2F) and colony formation (Figures 2N and S2G). Intracellular H_2_S accumulation as measured by flow cytometry was also enhanced in human GBM cells treated with FC9402 and HTS07545 (Figures 2O and S2H). The effects of inhibiting SQOR and rising H_2_S levels in turn, drove a compensatory response in which sulfide oxidation enzymes were found to increase over time (Figures S2I and S2J). These results indicate that sulfide oxidation and more specifically, the rate limiting enzyme SQOR, may serve as a therapeutic and mechanistic target in GBM.

### Mitochondrial dysfunction occurs in GBM cells with impaired sulfide oxidation capacity

To better understand how perturbation of sulfide oxidation affects GBM, we employed a combinatorial approach involving RNA sequencing, untargeted metabolomics, and flow cytometry in human HW1 GBM cells (Figure 3A). Following knockdown of SQOR, clear separation between control and experimental groups was observed (Figure S3A), with knockdown of SQOR having a profound effect on pathways related to mitochondrial function, oxidative phosphorylation, and nucleoside biosynthetic processes (Figure 3B). As disruption in sulfide oxidation was associated with increased mitochondrial related pathways, we reasoned that normal mitochondrial function was being impaired by accumulating H_2_S, leading cells to increase mitochondrial content as an adaptive response. Therefore, we measured mitochondrial content and intracellular H_2_S by flow cytometry using the fluorescent MitoTracker DeepRed and SF7-AM fluorescent probes. Augmented mitochondrial content (Figure 3C) was observed, with increased MitoTracker signal mirroring increased intracellular H_2_S levels following SQOR knockdown (Figure 3D). To determine the broader impact of insufficient sulfide oxidation resulting in upregulated mitochondria related pathways and increased mitochondrial content, we examined the overlap of differentially expressed genes (DEGs) following SQOR knockdown and described metabolic gene signatures^77^. This analysis revealed nucleotide, lipid, and cholesterol metabolism gene signatures to be elevated (Figure 3E and 3F). Interestingly, while many of these pathways begin with TCA/Krebs cycle metabolic intermediates, no changes were observed in expression of genes involved in the TCA cycle (Figure 3G). Conversely, knockdown of SQOR increased TCA/Krebs cycle intermediates citrate, α-ketoglutarate, succinate, fumarate, and malate (Figures 3H–3L). Accumulation of citrate from the TCA/Krebs cycle may also serve as a basis for increased fatty acid and cholesterol synthesis (Figure 3F). Additionally, SQOR knockdown induced a dramatic change in levels of amino acids (Figure S3B), including increased levels of glutamine, glutamate, and aspartate (Figures S3C–S3E) that also serve as substrates for *de novo* nucleotide synthesis (Figure 3F–3G). Likewise, the global increase in amino acids (Figure S3B) including sulfur containing methionine, cystine, and cysteine were found to be increased (Figures S3F–S3H). These results highlighted accumulation of numerous metabolic intermediates resulting from SQOR deficiency and prompted the hypothesis that diminished cellular fitness associated with SQOR silencing resulted from mitochondrial dysfunction and the inability to process these accreted energy substrates.

### Inadequate sulfide oxidation inhibits OXPHOS and leads to increased glycolytic demand in GBM

To test whether SQOR knockdown impaired mitochondrial function, we evaluated oxidative phosphorylation (OXPHOS) in human HW1 cells using the Seahorse XFe Mitostress test (Figures 4A–4E). Consistent with our RNA sequencing and metabolomics results, we observed decreased oxygen consumption rate (OCR) (Figure 4A), basal respiration (Figure 4B), and maximum respiration (Figure 4C) following SQOR knockdown. These results indicate that the ability to oxidize H_2_S is necessary for maintenance of mitochondrial respiration. The Mitostress test also indicated an increase in extracellular acidification rate (ECAR) (Figure 4D) and glycolysis (Figure 4E) with SQOR knockdown, highlighting an increased role for glucose as an energy source in GBM in the absence of SQOR. To validate these findings, we performed a colony formation assay in SQOR knockdown cells combined with the glycolysis inhibitor 2-Deoxy-D-Glucose (2-DG)^78^. SQOR deficient cells displayed an increased sensitivity to glycolysis inhibition with diminished growth and proliferation (Figure 4F).

Considering the results of SQOR knockdown on mitochondrial respiration, we confirmed our findings again using the pharmacological inhibitors, FC9402 and HTS07545. Evaluation of the SQOR inhibitor FC94902 (Figures 4G–4M and S4A–S4D), demonstrated robust inhibition of OXPHOS in HW1 cells. OCR was fully abrogated at concentrations as low as 0.1μM (Figure 4G), as was basal respiration (Figure 4H), and maximum respiration (Figure 4I). While ECAR (Figure 4J) and glycolysis (Figure 4L) were both found to be increased with FC9402 treatment. The decrease in mitochondrial respiration led to a decrease in ATP production necessary for fueling rapidly replicating tumor cells and an increase in ADP as represented as the ratio of ADP/ATP (Figure 4L). Meanwhile, SQOR inhibition forced cells to increase their reliance upon glycolysis which resulted in increased pyruvate (Figure S4A) and lactate secretion (Figure S4B). As was observed with SQOR knockdown, colony formation was further impaired by the combination of FC9402 and glycolysis inhibitor 2-DG (Figure 4M). Inhibition of SQOR with FC9402 also increased extracellular glutamine (Figure S4C), indicating accumulation of TCA cycle metabolic intermediates similar to what was observed with SQOR knockdown (Figure S3C). Notably, even though we detected altered mitochondrial activity, we did not find changes in electron transport chain proteins at the timepoints evaluated (Figure S4D). Testing the SQOR inhibitor, HTS07545 also yielded similar results (Figures S4E–S4M). OCR in HW1 cells treated with HTS07545 was decreased, albeit to a lesser extent (Figure S4E), as was basal respiration (Figure S4F), and maximum respiration (Figure S4G). Increases were also observed in ECAR (Figure S4H) and glycolysis (Figure S4I). Similarly, the ratio of ADP to ATP was increased (Figure S4J) indicating decreased energy production, while pyruvate (Figure S4K), lactate (Figure S4L), and extracellular glutamine (Figure S4M) were all increased. These results implicate SQOR in GBM cells as a protective measure against excessive H_2_S which otherwise may act as tumor suppressor via mitochondrial metabolic disruption.

### Lipids accumulate and diminish cancer stem cell phenotype in the absence of SQOR

The increase in citrate levels (Figure 3H), lipid, fatty acid, and cholesterol metabolic gene sets (Figure 3E–3G) following SQOR depletion suggested sulfide oxidation may regulate lipid metabolic activity in GBM cells. We therefore examined whether the accumulation of H_2_S, potentially disrupting mitochondrial function by inhibiting complex IV activity^58^ drove an increase in lipid synthesis (Figure 5A). Measurement of free glycerol and triglycerides, the storage form of fatty acids, was found to have an increased triglyceride to glycerol ratio in FC9402 treated HW1 cells (Figure 5B). Increased triglycerides coincided with a decrease in the ratio of oxidized NAD+ to reduced NADH (Figure 5C). These are important findings, as mitochondrial dysfunction leads to increased levels of the reducing equivalent, NADH, which feeds back into the TCA cycle to fuel conversion of citrate into lipids. Rather than accumulating free fatty acids, lipid synthesis results in triacylglycerol (TAGs) and cholesterol being stored in lipid droplets to prevent the effects of lipotoxicity. We therefore measured lipid droplet abundance by flow cytometry using the fluorescent lipid droplet probe, LipiDeep Red. Inhibition of SQOR with FC9402 was observed to coincide with a greater abundance of lipid droplets (Figure 5D). Due to the observed increase in TAGs and lipid droplets, we measured expression of key genes regulating the synthesis of lipids via qRT-PCR and observed TAG synthesis genes, *SREBF1*, *PPARG*, *SCD*, and *GPAT3* as well as cholesterol synthesis genes *SREBF2* and *HMGCR* to be increased following SQOR inhibition (Figure 5E and S5A). Interestingly, lipolysis genes involved in the oxidation of lipids were not altered (Figure S5B). There was also an increase in activation of enzymes involved in lipid synthesis observed via western blotting (Figure S5C). As we were expecting lipid synthesis to increase the abundance of stored TAGs to prevent lipotoxicity, we measured free fatty acids and did not observe differences between vehicle control or FC9402 treated cells (Figure S5D). With lipid synthesis capacity being increased following inhibition of SQOR without a clear signal for coordinated increases in lipolysis pathways, we performed a Seahorse XFe palmitate oxidation stress test (Figure 5F–H). HW1 cells were cultured under conditions of low glucose, sodium pyruvate, and glutamine either with vehicle control or the SQOR inhibitor FC9402. Cells were then provided sufficient amounts of either BSA-conjugate or BSA-conjugated palmitic acid. Under conditions of low glucose, sodium pyruvate, and glutamine, cells normally oxidize palmitic acid to fuel energy production. However, with SQOR inhibition we observed diminished ability for cells to utilize palmitic acid, and dampened OCR (Figure 5F), basal respiration (Figure 5G), and maximum respiration (Figure 5H). The reduced respiratory response observed in palmitate-supplemented media is consistent with impaired fatty-acid-supported respiration, although the current experiment does not distinguish this from the broader mitochondrial dysfunction caused by SQOR inhibition.

Since mitochondrial function is impaired with a decreased ability to oxidize lipids as an alternative fuel source and reliance upon glycolysis is limited, we examined the effect of SQOR inhibition on cancer stem cell (CSC) markers^38,66^. Nestin, SOX2, and Nanog protein levels were decreased with FC9402 treatment (Figure 5I). Additionally, measuring stem cell frequency by extreme limiting dilution assay (ELDA) with both pretreatment of L2 cells and treatment throughout the duration of the ELDA experiment, we observed a decrease in CSC frequency (Figures S5E–S5F and 5J–5K). Validation of findings with the SQOR inhibitor FC9402 was performed with HTS07545 (Figures S5G–S5K). HTS07545 drove a similar decrease in the ratio of NAD+ to NADH (Figure S5G), that fuels increased lipid droplet accumulation (Figure S5H). In addition, protein levels of CSC markers Nestin, SOX2, and Nanog were decreased (Figure S5I) which functionally decreased the abundance of CSCs with HTS07545 pretreatment and treatment at time of the experiment (Figures S5J–S5K). Interestingly, co-expression analysis of TCGA patient tumor transcriptional data indicates SQOR mRNA expression correlates with many triacylglycerol synthesis genes, including PPARG, GPAT3, and SREBF1 (Figures 5L–5N). An inverse relationship was observed between SQOR and cholesterol synthesis genes, including SREBF2, HMGCR, and HMGCS1 (Figures 5O–5Q). These findings suggest a complex relationship between SQOR and utilization of lipids, with active sulfide oxidation promoting enhanced metabolic flexibility in GBM to use both lipids and glucose as a fuel source. However, when sulfide oxidation is impaired, limitations to GBM metabolic flexibility render cells vulnerable to lipid accumulation and environmental nutrient stressors.

### SQOR loss renders GBM cells vulnerable to microenvironmental H_2_S

Due to the upregulation of SQOR observed in tumors from CGL TG mice (Figure 1E) as a means of eliminating tumor suppressive H_2_S in favor of fueling mitochondrial respiration, we performed a series of experiments involving the overexpression of CGL in human HW1 and SB2b glioma cells and treated with either FC9402 or HTS07545, followed by Seahorse XFe Mitostress test and colony formation assays (Figure 6A). Overexpression of CGL and enhanced H_2_S production capacity were confirmed by Western blotting and lead acetate assay, respectively (Figure 6B). Seahorse XFe Mitostress test measurement was performed on either the ORF control or CGL OE cells treated with FC9402 (Figures 6C–6F), and validation was conducted with HTS07545 (Figures S6A–S6E). The impact of FC9402 treatment on diminishing OCR in CGL OE cells was observed at a lower dose (0.01μM) as opposed to ORF in which the effects were observed at (0.1μM) (Figure 6C–6D), with basal respiration (Figure 6E), and maximum respiration (Figure 6F) significantly decreased in conditions of increased H_2_S. Functionally, CGL overexpression rendered the GBM cells more sensitive to SQOR inhibition with FC9402 and diminished colony formation and cellular growth (Figure 6G). We also observed increased ECAR (Figures 6H–6I) in CGL OE cells treated with lower doses of FC9402 and along with elevated glycolysis (Figure 6J). Validation of findings with HTS07545 demonstrated more complete response to SQOR inhibition at high doses than was observed in ORF control cells (Figures S6A–S6E). OCR was almost completely inhibited at 10μM concentrations in CGL OE cells, which was not observed in ORF controls (Figures S6A–S6B). Likewise, basal respiration was decreased (Figure S6C) and maximum respiration remained flat (Figure S6D) in the CGL OE cells treated with the SQOR inhibitor. Functionally, we observed increased sensitivity to HTS07545 in CGL OE cells as measured by decreased colony formation and cell growth (Figure S6E).

In reassessing the ability of SQOR inhibition to counter the GBM-supportive contributions of microenvironmental and systemic CGL activity, we tested FC9402 on CGL TG mice implanted with NPD glioma cells and compared to CGL TG mice treated with vehicle as well as CGL KO mice treated with vehicle (Figure 6K). FC9402 administration enhanced survival of the CGL TG mice compared to vehicle treated CGL TG mice and resulted in a similar survival curve as CGL KO mice (Figure 6K). These combined *in vitro* and *in vivo* results indicate that SQOR is a necessary means by which GBM cells defend and support themselves against cell-intrinsic and/or tumor microenvironmental H_2_S.

## Discussion

Our findings identify sulfide oxidation as a critical metabolic defense program in GBM and establish SQOR as a key determinant of tumor metabolic fitness. While sulfide metabolism has increasingly been implicated in cancer biology^52,53,55,79,80^, its role in GBM has remained difficult to reconcile because the transsulfuration pathways and CGL activity that support cysteine availability, glutathione synthesis, and redox buffering also generate H_2_S, a metabolite with both tumor-promoting and tumor-suppressive effects in GBM^32–35,38,41,43,71^. Our data provide a framework to resolve this paradox by showing that GBM upregulates sulfide oxidation capacity, particularly through SQOR, to buffer the liability imposed by endogenous and/or microenvironmental H_2_S. In this context, SQOR preserves mitochondrial respiration, maintains access to lipid fuel under stress, and promotes cancer cell growth. Together, these findings support a model in which SQOR is not simply a detoxification enzyme, but a metabolic safeguard that enables GBM cells to exploit sulfide metabolism without incurring sulfide toxicity as depicted in the Figure 7 model.

Several lines of evidence support this model. GBM exhibited elevated sulfide oxidation capacity that was further induced by exogenous H_2_S (Figures 1E, 1G–1I, and S1C), indicating that this pathway is both basally engaged and adaptively mobilized in response to sulfide stress. SQOR and ETHE1 expression also increased with glioma grade (Figures 1J–1M), consistent with progressive selection for sulfide disposal during malignant evolution. Functionally, genetic or pharmacologic SQOR inhibition suppressed proliferation (Figures 2F, 2N, S2B, and S2F) and clonogenic growth (Figures 2H, 2M, 2C, and 2G), while it extended survival in orthotopic models (Figures 2D, 2E, 2K, and 2L), identifying sulfide oxidation as a metabolic dependency in GBM. Notably, this dependency intensified with sulfide burden, as CGL overexpression increased H_2_S-generating capacity and sensitized cells to SQOR inhibition (Figures 6G and S6E), arguing that sulfide levels set the threshold for SQOR requirement.

Mechanistically, our data identify mitochondrial sulfide toxicity as the central consequence of SQOR loss. As the first and rate-limiting enzyme in mitochondrial sulfide oxidation, SQOR sits at the interface of sulfide detoxification and electron transport by channeling sulfide-derived electrons into the coenzyme Q pool^58,61^. Loss of SQOR therefore not only permits intracellular and systemic H_2_S accumulation^59,60^ (Figures 2O, S2D,S2E, and S2H) which can lead to potential anti-GBM persulfidation^38^, but also disables a key route for mitochondrial sulfide clearance. In this setting, respiration collapsed (Figures 4A, 4G, and S4E), with reductions in oxygen consumption, basal respiration (Figures 4B, 4H, and S4F), and maximal respiratory capacity (Figures 4C,4I, and S4G). These findings are consistent with the known ability of excess H_2_S to inhibit mitochondrial complex IV, destabilize respiratory supercomplexes, and interfere with oxidative phosphorylation^81,82^. The accompanying increase in mitochondrial gene expression and mitochondrial content observed after SQOR depletion (Figures 3B and 3C) may therefore represent a compensatory response to impaired respiratory efficiency rather than improved mitochondrial function^83^. In this context, elevated mitochondrial biogenesis or retention would reflect an attempt to preserve ATP output despite sulfide-mediated respiratory stress.

A prominent consequence of SQOR loss was the accumulation of TCA cycle intermediates without a corresponding increase in canonical TCA cycle gene expression (Figure 3G), consistent with prior studies implicating SQOR in the regulation of redox circuitry^84,85^. This pattern argues against transcriptional rewiring of central carbon metabolism and instead points to a functional bottleneck in mitochondrial oxidative activity. Citrate, α-ketoglutarate, succinate, fumarate, and malate accumulated following SQOR depletion (Figures 3H–3L), consistent with impaired consumption of TCA-derived carbon under conditions of respiratory insufficiency. The accompanying increase in glutamine and glutamate further supports a model in which anaplerotic input persists but cannot be efficiently oxidized. Rather than being fully catabolized for ATP generation, these intermediates appear to be diverted into biosynthetic or storage pathways as has previously been linked to altered NAD(P)H pools^86^. This metabolic congestion likely contributes to the increased nucleotide, lipid, and cholesterol signatures observed in transcriptomic analyses (Figures 3E and 3F) and highlights SQOR as an important regulator of TCA cycle throughput rather than simply mitochondrial sulfide detoxification.

The bioenergetic consequences of SQOR deficiency in our GBM lines were partially buffered by a shift toward glycolysis. As mitochondrial oxidative phosphorylation and ATP generation are greatly hampered with a loss of SQOR, reliance on glycolysis for cellular survival is paramount^87^. Both SQOR knockdown and pharmacologic inhibition increased ECAR and glycolytic dependence (Figures 4D, 4E, 4J, 4K, S4H and S4I), and SQOR-deficient cells were more sensitive to 2-deoxyglucose (Figures 4F and 4M). These results indicate that GBM cells attempt to compensate for impaired oxidative phosphorylation by increasing glucose utilization. However, this compensation was incomplete, as ATP levels were decreased (Figures 4L and S4J). This is an important point in the context of GBM biology, where metabolic plasticity and dynamics are defining features of tumor survival in the nutrient- and oxygen-limited brain microenvironment^66,88–90^. Our data support a model in which SQOR underwrites metabolic plasticity by preserving mitochondrial competence: when sulfide oxidation is disrupted, GBM cells can transiently compensate by increasing glycolysis, but they lose the mitochondrial capacity required to reengage oxidative metabolism and utilize alternative fuel sources.

One of the most striking downstream effects of SQOR inhibition was the remodeling of lipid metabolism. Increased citrate abundance (Figure 3H), reduced NAD+/NADH ratio (Figures 5C and S5G), induction of lipogenic transcriptional programs (Figures 3F, 3E, and S3B), elevated triglyceride content (Figure 5B), and accumulation of lipid droplets (Figures 5D and S5H) together support a model in which impaired sulfide oxidation redirects carbon into lipid synthesis and storage. This response is likely driven by mitochondrial dysfunction: when electron transport is compromised, NADH accumulates, TCA-cycle intermediates accumulate, and citrate becomes available for export into the cytosol to support fatty acid and sterol synthesis. Under these conditions, excess lipids are stored as triacylglycerol within lipid droplets, presumably to limit lipotoxicity. However, this increase in lipid storage did not translate into improved metabolic resilience. Instead, SQOR inhibition suppressed palmitate oxidation and reduced the ability of GBM cells to mobilize lipid stores as an alternative energy source (Figures 5F–5H). These data suggest that sulfide oxidation is required not only to prevent toxic sulfide accumulation but also to maintain lipid catabolism as a stress-adaptive fuel pathway.

This distinction between lipid storage and lipid utilization is particularly relevant in GBM, where fatty acid oxidation has been implicated in survival during nutrient limitation and in maintenance of stem-like states^66^. In our study, SQOR inhibition caused lipid droplets to accumulate while simultaneously diminishing fatty acid oxidation, indicating that cells become trapped in a metabolically inflexible state. Carbon is diverted into neutral lipid storage but cannot be efficiently reclaimed to support mitochondrial ATP production. This phenotype likely reflects the broader collapse of mitochondrial function caused by sulfide accumulation. It also provides a mechanistic explanation for the observed reduction in stem cell markers (Figures 5I and S5I) and stem cell frequency (Figures 5J, 5K, S5E, S5F, S5J, and S5K) after SQOR inhibition. Glioblastoma stem-like cells are thought to depend on metabolic adaptability to survive therapeutic and microenvironmental stress^38,66,91,92^. By impairing both oxidative phosphorylation and lipid catabolism, SQOR targeting may selectively undermine the metabolic programs that sustain aggressive cell growth and regeneration.

These mechanistic insights also carry scientific and translational implications. They identify SQOR as a targetable metabolic vulnerability in a disease with few tractable dependencies and indicate that tumors with high sulfide oxidation capacity may be especially susceptible to SQOR inhibition. Expression of transsulfuration enzymes such as CGL or CBS, or direct measures of sulfide metabolite burden, may therefore help define responsive tumors. Furthermore, the future use of dietary, hormonal, and/or pharmacological interventions that boost endogenous H_2_S and persulfidation^44,68,93–97^ or utilize exogenous sulfide sources^98–101^ in combination of SQOR inhibitors for the treatment of GBM patients should consider the SQOR status of their tumors to ensure patient safety and therapy efficacy. Likewise, the wide spectrum of sulfide production and/or oxidation enzyme levels discovered in our panel of human and murine GBM cell models sheds light on the heterogenous nature of GBM, with both researchers and clinicians needing to take these factors into context for future studies and when reevaluating past results. The metabolic state induced by SQOR inhibition also suggests novel rational combination strategies, such that increased glycolytic reliance under SQOR inhibition may create sensitivity to glycolysis-targeting approaches, whereas impaired lipid utilization may expose liabilities related to lipid overload, peroxidation, or ferroptotic stress. More broadly, because sulfide metabolism intersects with glutathione synthesis and redox homeostasis, therapies that induce SQOR inhibition may sensitize GBM to currently used radiation and chemotherapeutic standards of care.

Taken together, these data place sulfide oxidation within the core logic of GBM metabolic adaptation. Sulfur metabolism in GBM is intrinsically double-edged: it supports anabolic and redox demands, but in doing so generates a mitochondrial poison. SQOR resolves this tradeoff by converting sulfide disposal into a mitochondrial support function, thereby preserving respiration, preventing accumulation of TCA-intermediates, and promoting access to alternative fuels. When this buffering system is removed, the tumor is not simply detoxification-deficient; it becomes metabolically fragile. Mitochondrial respiration falters, metabolic intermediates accumulate, lipid reserves become unusable, and glycolysis cannot fully compensate. SQOR is therefore not peripheral to GBM metabolism, but a central node that converts sulfide stress into metabolic fitness. Targeting this node provides a mechanistically grounded strategy to collapse the adaptive flexibility that underlies GBM aggressiveness.

### Limitations of the study

Although the present data establish a tumor-intrinsic role for SQOR in maintaining mitochondrial fitness, they do not fully resolve how sulfide metabolism is shaped by the broader brain microenvironment. Astrocytes, immune populations, and vascular cells are all likely to participate in sulfur exchange and signaling, and these interactions may influence both tumor behavior and therapeutic response *in vivo*^71^. In addition, while SQOR inhibition consistently increased intracellular H_2_S, the downstream sulfur species that mediate the observed phenotypes remain undefined. H_2_S, persulfides, and polysulfides are likely to have distinct effects on mitochondrial enzymes, redox signaling, and lipid metabolism. Defining these species, together with the relevant protein persulfidation events, will be important for sharpening the mechanistic framework that emerges from this study. Finally, translation of these findings will require a clearer understanding of the pharmacologic properties of small-molecule driven SQOR inhibition in the brain. In particular, selectivity, pharmacokinetics, and brain penetrance will need to be established to determine whether this pathway can be exploited safely and effectively in GBM.

## Methods

### Resource availability

#### Lead contact

Requests for information, resources, and reagents should be direct to and will be fulfilled by lead contacts, Justin D. Lathia (lathiaj@ccf.org) and Christopher Hine (hinec@ccf.org).

#### Materials availability

Materials and reagents generated during this study are available upon request from Christopher Hine (hinec@ccf.org) after a materials transfer agreement has been completed.

See the Key Resources Table and **Oligo Sequences Table** for additional information for reagents, antibodies, oligo sequences, and/or resources used in this study.

#### Data and code availability

- This paper does not report original code
- Original western blot images, H_2_S production images, metabolomics data, RNAseq data, and source data will be included as Supplementary Data files and/or deposited on a publicly available data sharing platform prior to the date of publication. Metabolomics data from this study is currently deposited on the NIH Common Fund's National Metabolomics Data Repository (NMDR) website, the Metabolomics Workbench, https://www.metabolomicsworkbench.org where it has been assigned Study ID ST005168. The data can be accessed directly via its Project DOI: http://dx.doi.org/10.21228/M8M587. RNAseq data has been uploaded to NCBI GEO at GEO ID: GSE346875.
- Any additional information required to reanalyze the data reported in this paper is available from the lead contact(s) upon request.

### Experimental model and subject details

#### Mice

All studies involving the use of animal subjects were performed in accordance with Cleveland Clinic Institutional Animal Care and Use Committee (IACUC) approved protocols (Protocol#00003408). All animals were housed in specific pathogen-free facilities maintained by the Cleveland Clinic Biological Resource Unit, under 12-hour light:12-hour dark cycles. Room conditions were maintained between 20–23°C, and 30–70% relative humidity, with *ad libitum* access to food and water. Unless noted, mouse experiments were performed on C57BL/6J mice (RRID:IMSR_JAX:000664) which were ordered and delivered between 6–8 weeks of age for experimental use. Experimental whole body CGL knockout (KO) mice^67,68^ and whole body CGL wildtype transgenic (TG) overexpressing mice that contain two to four copies of the CGL gene under its endogenous promoter^69,70^ were generated as previously describe and bred and backcrossed to C57BL/6J at Cleveland Clinic and weaned between 21–25 days of age. At the time of weaning, male and female mice were separated into cages with littermates containing up to 5 mice per cage. Ear markings were used for identification and genotyping purposes. CGL KO and CGL TG mice were used for experimental purposes when they were between 6–8 weeks of age.

#### Cell culture

Patient derived HW1, SB2b, WK1, RN1, PB1, and MMK1 GBM cells were previously isolated and described^102–104^. Derivation and characterization of patient derived L0, L1, and L2 GBM cells was previously described^105,106^. T3832^107^, T3691^107^, T4121^108^, hGBM23^38^ and DI318^109,110^ patient derived GBM cells were generated and reported previously Murine glioma models, KR158^72^ and NPD^73,74^ used extensively in this study were generated and characterized previously. KR158 (NF1^−/−^ and p53^−/−^) cells were derived from a spontaneously occurring astrocytoma and NPD (shp53, NRA, and PDGF cells were generated using the Sleeping Beauty transposon system. Additional murine glioma models employed in the study were CT-2A^111^, SB28^112^, GL261^113^, CPA^114^, OL61^115^, mGBM6, and fGBM7. Human glioblastoma and murine glioma cells were cultured and maintained under stem cell enriching conditions in N_2_ complete media consisting of DMEM/F-12 medium supplemented with recombinant human epidermal growth factor (rhEGF), recombinant human fibroblast growth factor (rhFGF), N-2 supplement, and penicillin/streptomycin. Human L2 cells were cultured in complete neurobasal media consisting of Neurobasal medium supplemented with rhEGF, rhFGF, B27 supplement, penicillin/streptomycin, sodium pyruvate, and glutamine. Adherent cell culture was performed using geltrex-coated tissue culture flasks and dishes. Non-adherent cell culture was performed using non-tissue culture treated dishes and ultra-low adherence multi-well plates. For all cell models, complete media was replaced every other day, and cells were passaged at 85–90% confluency. Passaging of cells was performed using Accutase. Cells were maintained at 37°C with 5% CO_2_.

### Method details

#### Intracranial tumor implantation

Prior to intracranial tumor implantation, mice were anesthetized by inhalation of anesthetic (2–2.5% isoflurane). Mice were then secured using a stereotactic apparatus and intracranially injected with 10μL of glioma cells suspended in DMEM/F-12 medium. Cell number used for experiments were as follows: KR158, 50,000 cells per mouse; NPD, 2,500 cells per mouse. Stereotactic injection of glioma cells was targeted to the left hemisphere approximately 0.5mm rostral and 1.8mm lateral to the bregma with a depth of 3.5mm from the scalp. Using these coordinates, injections avoid direct contact with the hypothalamus. Following injection of cells, the needle was maintained in its place for an additional 60 seconds to avoid backflow of cell suspension. The needle was then retracted slowly. Afterwards, mice were monitored daily to monitor for signs of neurological and behavioral abnormalities, indicative of the presence of a tumor.

#### Intraperitoneal injections

In experiments involving treatment with the SQOR inhibitor, FC9402. Tumor were implanted and male or female C57BL/6J mice began receiving treatments 3 times each week administered by intraperitoneal (IP) injection following similar approaches reported for other solid tumor studies^79,80^. Stock FC9402 solution was prepared by resuspending the compound at 10mg/mL in sterile DMSO. Solution was stored at −20°C until ready for use within 6 months. FC9402 was further diluted to 0.5mg/kg in 40% PEG400, 1% Tween-80, and 59% saline. An equal volume of DMSO was diluted in the same buffer to prepare the matching vehicle control. Both FC9402 and vehicle control were sterile filtered through a 0.22μm syringe filter and syringes were prepared for IP injections. Treatments continued until mice reached endpoint. Mice intended for metabolomics sample preparation were given a single injection of ketamine/xylazine at endpoint prior to saline perfusion.

#### Tissue preparation and LCMS analysis

At indicated time points, control and tumor bearing mice were IP injected with ketamine/xylazine. Mice were then perfused mice with 10mL of sterile saline. Tumor and contralateral non-tumor brain tissue, along with tissue from non-tumor bearing, control mice were snap frozen and stored at −80°C until processed for untargeted metabolomics. Untargeted metabolomics of control tissues as well as tumor and non-tumor brain hemispheres were done using Bruker Daltonics timsTOF pro 2 mass spectrometer coupled with Agilent 1290 Infinity 1290 II LC system. Reverse phase chromatography was performed by injecting 6μL of each sample on a Thermo Accucore Vanquish C18 column with dimensions 100 × 2.1mm, 1.5μm particle size (Thermo P/N 27101–102130) at 60°C coupled to an Agilent 1290 Infinity II UHPLC by gradient elution with mobile phase A (0.1% formic acid in water) and mobile phase B (0.1% formic acid in acetonitrile). After LC separation, metabolites were detected using Bruker Daltonics timsTOF pro 2 mass spectrometer. Ionization was done using vacuum insulated probe heated electrospray ionization source ion source in positive and negative ionization mode in separate runs. The end plate offset was set to 500 V, and the capillary voltage was set to 4,500 V in both positive and negative modes. Nitrogen was used as dry gas at a flow rate of 8L/min and the nebulizer gas was set at 2.0 bar. The dry temperature was 230°C and the sheath gas temperature was 400°C, using a sheath gas flow of 4L/min. PASEF scan mode was used covering a mass range of 20–1,300 m/z. Active exclusion was set to 0.1 min. The exclusion window was 0.010 m/z and 0.010 Vs/cm2. The acquisition cycle was 100ms (with a mobility scan range of 0.10–1.50 Vs/cm2. The Ramp rate was 9.08 Hz and the number of PASEF ramps was set to 2 resulting in a cycle time of 0.32 s. The collision energy was set to fixed values of 50 eV and 20 eV in positive and negative modes.

Data was processed using MetaboScape 2025b. Mass and mobility recalibration was done using sodium formate and Agilent Tune mix, respectively, which were inserted in defined calibration segment of each measurement. Metabolite identification was done using MetaboScape 2025b utilizing a Bruker NIST 2020 MSMS Spectral library, rule-based lipid annotation and publicly available spectral libraries from MassBank of North America (MoNA) containing 13,303 unique compounds (positive mode) and MSDIAL Metabolomics MSP Spectral Kit containing 12,879 unique compounds (negative mode) with 80% identification cut-off score.

Pathway enrichment analysis was performed using MetaboAnalyst 5.0 to identify top 25 altered pathways for putatively identified metabolites in the dataset. In this analysis the Pathway-associated metabolite sets (SMPDB) database, which consists of 99 pathways from the Small Molecule Pathway Database 5 was used to map the putatively identified ions to various pathways.

#### Lentivirus knockdown

Generation of stable shCtrl or SQOR knockdown cells was performed using prepackaged lentiviral particles (Sigma-Aldrich), and open reading frame (ORF) vector control or CGL overexpressing (CGL OE) cells were generated using prepackaged lentiviral particles (Vector Builder). MISSION pLKO.1-puro non-target shRNA control transduction particles were used to generate shCtrl cells. Knockdown of SQOR (sequence NM_021199) in human glioblastoma cells was performed with 3–5 individual MISSION shRNAs ordered as prepackaged lentiviral particles for each experiment. Knockdown of SQOR (sequence NM_021507) in murine glioma cells was performed with 3–4 individual MISSION shRNAs ordered as prepackaged lentiviral particles for each experiment. Vector Builder ORF control (pLV[Exp]-Puro-EF1A>ORF_Stuffer (VB010000–9667qqs) and CGL OE (pLV[Exp]-Neo-EF1A>hCTH[NM_001902.6] (VB260108–1269bkn) vectors were used to generate stable ORF and CGL OE cells. Generation of stable cell lines for experimental use was performed by seeding cells at 2.5 × 10^6^ cells per well of a 6-well plate. The following day, cells were infected at an MOI=10 for SQOR KD experiments, and MOI=5 for CGL OE experiments in 1mL of complete media containing 8ug/mL polybrene. Cells were incubated with lentiviral particles for 8–12 hours. Virus containing media was replaced with complete media for 24 hours to ensure sufficient time for shRNA expression. Selective antibiotic was then added and maintained for 5–7 days until non-transduced cells were killed off. Puromycin concentrations used: KR158, 4μg/mL; NPD, 2μg/mL; HW1, 0.4μg/mL; SB2b, 0.5μg/mL; L2, 2.0μg/mL. Neomycin concentrations used: HW1, 300μg/mL; SB2b, 400μg/mL. Cells were then expanded and used for experiments.

#### Tissue and cell preparation for western blotting

Snap frozen tissue and cell pellets were thawed on ice. Samples were prepared in RIPA lysis buffer containing protease and phosphatase inhibitor cocktails. Tissue homogenization was performed using a handheld homogenizer. Cell pellets were resuspended with pipetting. Samples were incubated for 30 minutes on ice with vortexing at regular intervals. Insoluble material was removed by centrifuging samples for 10 minutes at 10,000 × g and 4°C. Soluble fraction was transferred to a new tube, and protein quantification was completed using the protein assay kit (BioRad) read on a SpectraMax iD3 Multi-Mode Plate Reader (Molecular Devices). Protein concentrations were normalized and 15–25μg of protein were loaded for each sample on Criterion gels and transferred to PVDF membranes for western blotting. Antibodies used for blots are listed in the Antibody Key Resources Table. Uncropped blot images are presented in Supplemental Material files.

#### Tissue and cell preparation for lead acetate assay

H_2_S production from tissue and cell extracts were measured using the lead acetate/lead sulfide method^116^. Snap frozen tissue and cell pellets were thawed on ice. Samples were prepared in passive lysis buffer. Tissue homogenization was performed using a handheld tissue homogenizer, while cell pellets were resuspended with vigorous pipetting. Samples were incubated for 30 minutes on ice with vortexing at regular intervals. To ensure complete sample lysis, tissue and cell homogenates were snap frozen using liquid nitrogen and thawed on ice 3 times. Insoluble material was removed by centrifuging samples for 10 minutes at 10,000 × g and 4°C. Soluble fraction was transferred to a new tube, and protein quantification was completed using the protein assay kit (BioRad) read on a SpectraMax iD3 Multi-Mode Plate reader (Molecular Devices). Protein concentrations were normalized to between 2–8μg/uL of protein. Between 50 and 100μg of protein were combined in 150μL total reaction mixture containing 10mM L-cysteine and 1mM pyridoxal 5’-phosphate (PLP) in PBS. The complete reaction mixture containing tissue or cell homogenates was prepared in a 96-well plate. A filter paper embedded with lead acetate was placed on top of the assay plate, then the reaction was incubated at 37°C between 1 and 24 hours.

#### Tissue preparation for H_2_S measurement

Plasma H_2_S levels were measured using the fluorescent SF7-AM probe. Snap frozen plasma samples were thawed on ice. Samples were then diluted at a 1:3 ratio in PBS containing 10μM SF7-AM. Samples were assayed in black walled, clear bottomed 96-well plates. After 30 minutes of incubation at room temperature in the dark, fluorescence was measured using a SpectraMax iD3 Multi-Mode Plate Reader (Molecular Devices), Excitation: 498nm, Emission: 526nm.

#### Extraction of mRNA for qRT-PCR

Human GBM cells were cultured in 12-well plates and treated in triplicate with SQOR inhibitor, FC9402 at indicated concentrations and for specified durations of time. Cells were then collected, snap frozen, and stored at −80°C until processing. Total RNA was isolated using the mRNA extraction kit (Qiagen) following the standard protocol with DNase treatment. Concentrations of mRNA were measured using a NanoDrop UV-Vis spectrophotometer (Thermo Fisher Scientific). cDNA was synthesized using the Verso cDNA Reverse Transcription kit (Thermo Fisher Scientific). qRT-PCR reactions were performed using SYBR green universal master mix (Applied Biosystems) with primers ordered from Integrated DNA Technologies on an Applied Biosystems StepOne Real-Time PCR System (Applied Biosystems). Threshold cycle (*C*_*t*_) values for each target gene normalized to the expression levels of *Actin*, with relative expression calculated using the ΔΔ*C*_*t*_ method.

#### Bulk RNA sequencing

Knockdown and selection of SQOR was performed in HW1 human GBM cells. Following selection, cells were passaged and expanded as triplicates in 10cm tissue culture dishes. Cells were then collected, snap frozen, and stored at −80°C until proceeding with RNA extraction. Total RNA was isolated using the mRNA extraction kit (Qiagen) following the standard protocol with DNase treatment. Concentrations of RNA were measured using a Qubit RNA HS Assay kit and Qubit fluorometer according to the manufacturer’s instructions. The RNA integrity number (RIN) for each sample was measured using an Agilent 2200 TapeStation. RNA-seq libraries were generated using Illumina Total RNA Prep with Ribo-Zero Plus (Cat. 20040525). Sequencing libraries were sequenced on the NovaSeq 6000 System at the Microbial Sequencing & Analytics Resource (MSAAR) facility at Cleveland Clinic Research Laboratories. Reads were trimmed of low-quality reads and adapter sequences using Trimmomatic (version 0.32). Quality filtered reads were mapped to the human genome (version GRCh38.p14), using STAR (version 020201). Transcripts per million (TPM) and expected counts for each gene were calculated using RNAnorm (https://github.com/Lyd0527/RNAnorm). Differential expression analysis was conducted using the Bioconductor package edgeR (version 3.22.3) using the decide Tests DGE function. Functional enrichments of gene ontology (GO) categories were performed using topGO package in R with Bonferroni-corrected P-values < 0.05 taken as significant.

#### Cell preparation for MS analysis

Knockdown and selection of SQOR was performed in HW1, human GBM cells. Following selection, cells were passaged and expanded as 6 replicates in 10cm tissue culture dishes. Cells were detached from the tissue culture dish using Accutase, washed with PBS, and resuspended in PBS to count. Sufficient volume needed for 1 × 10^6^ cells/sample were transferred to individual cryotubes and centrifuged at 400 × g for 5 minutes. Supernatants were removed, the cell pellets were snap frozen, and samples were stored at −80°C until performing LC-MS/MS analysis.

Metabolic quenching, lysis, metabolite and lipid extraction was performed by adding 1.0mL ice cold 80% aqueous methanol containing 10μM each deuterated (D_3_)-creatinine, (D_3_)-alanine, (D_4_)-taurine, (D_3_)-lactate (Sigma-Aldrich) and 10μL LipidSplash deuterated lipid internal standards (Avanti Polar Lipids. Alabaster, AL) to each sample. Samples were homogenized using MP Bio Matrix A (garnet/ceramic sphere) and subjected to a Folch extraction. Samples were rested on ice for 10 minutes before phase separation via centrifugation at 2500 × g for 15 minutes. 1mL of the aqueous phase was removed and dried to completion under nitrogen gas before being resuspended in 100μL water. 1.5mL of the organic phase was dried to completed under nitrogen gas and resuspended in 100μL 1:1 acetonitrile:isopropanol. 3μL of sample was subjected to online LC-MS analysis.

Polar metabolite analyses were performed by untargeted LC-HRMS. Briefly, Samples were injected via a Thermo Vanquish UHPLC and separated over a reversed phase Thermo HyperCarb porous graphite column (2.1×100mm, 3μm particle size) maintained at 55°C. For the 20-minute LC gradient, the mobile phase consisted of the following: solvent A (water / 0.1% FA) and solvent B (ACN / 0.1% FA). The gradient flowing at 300μL/min was the following: 0–1min 1%B, increase to 15%B over 5 minutes, continue increasing to 98%B over 5 minutes, hold at 98%B for five minutes, re-equilibrate at 1%B for five minutes. The Thermo IDX tribrid mass spectrometer was operated in both positive and ion mode, scanning in ddMS^2^ mode (2 μscans) from 70 to 800 m/z at 120,000 resolution with an AGC target of 2e5 for full scan, 2e4 for ms^2^ scans using HCD fragmentation at stepped 15,35,50 collision energies. Source ionization setting was 3.0 and 2.4kV spray voltage respectively for positive and negative mode. Source gas parameters were 35 sheath gas, 12 auxiliary gas at 320°C, and 8 sweep gas. Calibration was performed prior to analysis using the Pierce^™^ FlexMix Ion Calibration Solutions (Thermo Fisher Scientific). Integrated peak areas were then extracted manually using Quan Browser (Thermo Fisher Xcalibur ver. 2.7). Untargeted differential comparisons were performed using Compound Discoverer 3.3 (Thermo Fisher) to generate a ranked list of significant compounds with tentative identifications from BioCyc, KEGG, and internal compound databases. Purified standards were then purchased and compared in retention time, m/z, along with ms2 fragmentation patterns to validate the identity of significant hits.

#### Extracellular lactate, pyruvate, and glutamine measurement

Cells were seeded at 10,000 cells/well of a 96-well plate in 100μL of N2_C_ media. The following day, SQOR inhibitors, FC9402 and HTS07545 or vehicle control were added to their respective wells at the concentrations indicated. Cells were incubated with the SQOR inhibitors for the time periods indicated, at which point media was collected for relative measurement of extracellular metabolite levels. Standard assay procedures were followed according to manufacturer instructions for Lactate-Glo (Promega), Pyruvate-Glo (Promega), and Glutamine/Glutamate-Glo (Promega) assays. Cell culture media was diluted 1:100 for lactate and glutamine/glutamate assays in PBS, and 1:20 for pyruvate assays. Luminescence readings were recorded on a SpectraMax iD3 Multi-Mode Plate reader (Molecular Devices).

#### Intracellular triglyceride and NADH measurement

Cells were seeded at 10,000 cells/well of a white walled, clear bottom, 96-well plate in 100μL of N2_C_ media. The following day, SQOR inhibitors, FC9402 and HTS07545 or vehicle control were added to their respective wells at the concentrations indicated. Cells were incubated with the SQOR inhibitors for the time periods indicated, at which point standard assay procedures were followed according to manufacturer instructions for Triglyceride-Glo (Promega) and NAD/NADH-Glo (Promega) assays. Luminescence readings were recorded on a SpectraMax iD3 Multi-Mode Plate reader (Molecular Devices).

#### Measurement of ADP/ATP

Cells were seeded at 10,000 cells/well of a white walled, clear bottom, 96-well plate in 100μL of N2_C_ media. The following day, SQOR inhibitors, FC9402 and HTS07545 or vehicle control were added to their respective wells at the concentrations indicated. Cells were incubated with the SQOR inhibitors for the indicated time periods, at which point standard assay procedures were followed according to manufacturer instructions for the ADP/ATP ratio assay kit (Dojindo). Luminescence readings were recorded on a SpectraMax iD3 Multi-Mode Plate reader (Molecular Devices).

#### Free fatty acid measurement

Cells were seeded at 250,000 cells/well of a 6-well plate in 2 mL of N2_C_ media. The following day cells were treated with either vehicle control or FC9402 at the concentrations indicated. Cells were incubated for the time periods indicated, at which point standard assay procedures were followed according to manufacturer instructions for the free fatty acid assay kit (Abcam). Fluorescence readings, Exc: 535 nm and Emm: 587 nm, were recorded on a SpectraMax iD3 Multi-Mode Plate reader (Molecular Devices).

#### Flow cytometry

Cells were seeded at 100,000 cells/well of a 12-well plate in 1 mL of N2_C_ media. Experiments involving the use of SQOR inhibitors, FC9402 or HTS07545, the treatment or vehicle controls were added the following day. Cells were incubated for the indicated periods of time, at which point flow cytometry was performed. In preparation for flow cytometry, cells were washed 3 times with pre-warmed HBSS. Fluorescent probes, SF7-AM (1 μM) for H_2_S detection, Lipi-Deep Red (0.1 μM) for lipid droplet labeling, and MitoTracker Deep Red (250 nM) for labeling mitochondria were prepared in pre-warmed HBSS. Fluorescent probes were added to cells and incubated at 37°C for 30 minutes. Cells were then washed 3 times with pre-warmed PBS and incubated for 5 minutes with Accutase to dissociate cells. PBS was used to resuspend cells, followed by centrifugation for 5 minutes at 400 × g. Supernatants were decanted and cells were resuspended in FACS buffer (0.2% BSA in PBS) with live/dead Fixable Blue viability dye. Cells were incubated at room temperature protected from light for 30 minutes. Cells were washed 1 time with PBS and resuspended in FACS buffer for flow cytometry acquisition on a LSR Fortessa instrument with FACSDiva software (BD Biosciences), analyzed using FlowJo software (v.10, BD Biosciences).

#### Seahorse assays

The Seahorse XFe96 Analyzer (Agilent) was used to measure oxygen consumption rate (OCR) and extracellular acidification rate (ECAR). Assay procedures followed standard manufacturer instructions. Cells were seeded at 10,000 cells/well in 100 μL of N2_C_ media at which point they were allowed to settle and adhere prior to returning to incubator overnight at 37°C. The following day, treatments including the vehicle control or SQOR inhibitors, FC9402 and HTS07545 were added at the desired final concentration in an additional 100 μL of N2_C_ media for the MitoStress test. The Palmitate Oxidation Stress test was performed by removing the N2_C_ media and replacing it with substrate-limited growth media (Seahorse XF media supplemented with 0.5 mM glucose, 1.0 mM L-glutamine, and 0.5 mM L-Carnitine) containing either vehicle control or SQOR inhibitors, FC9402 and HTS07545. The day of the MitoStress test, media was replaced with Seahorse XF media supplemented with 1 mM sodium pyruvate, 2 mM L-glutamine, and 20 mM glucose. The palmitate oxidation stress test was performed by replacing the substrate-limited growth media with assay media consisting of Seahorse XF media, 2 mM glucose, and 0.5 mM L-Carnitine. Concentrations of inhibitors for the MitoStress test were as follows: Oligomycin=1 mM, FCCP=1 mM, Rotenone/Antimycin A=0.5 mM/0.5 mM, and 2-Deoxy-D-glucose=1 μM. Palmitate oxidation stress test was performed by immediately prior to assay, BSA-conjugate or BSA-conjugated palmitic acid were added. Concentrations of inhibitors for the palmitate oxidation stress test were as follows: Etomoxir=4 μM, Oligomycin= 1.5 μM, FCCP= 1 μM, and Rotenone/Antimycin A=0.5μM/0.5μM. OCR and ECAR values were normalized to cell count performed using Hoechst staining imaged on a Cytation 5 instrument.

#### Colony formation assay

Cells were seeded at the indicated concentrations: HW1, 5,000 cells/well; SB2b, 10,000 cells/well; KR158, 2,000 cells/well in a 12-well plate. Media changes were performed every other day for 14–21 days depending upon speed of colony formation. SQOR inhibitors, FC9402 and HTS07545, and 2-Deoxy-D-Glucose (2-DG) were added at concentrations indicated 24 hours after cell seeding and were refreshed every other day for 14–21 days along with regular media changes. At experimental endpoint, media was removed and cells were washed 3 times with PBS. Cells were fixed and permeabilized with ice cold 100% methanol. Subsequent staining was performed for 30 minutes at room temperature with a 0.5% crystal violet solution containing 25% methanol and 75% DI H_2_O. The crystal violet solution was removed, and the tissue culture plates were washed repeatedly in H_2_O. Tissue culture plates were scanned using an EPSON Scanjet (HP).

#### Incucyte live cell imaging

Cells were seeded at 5,000 cells/well in a 24-well plate. 24 hours after seeding cells, tissue culture plates were inserted into an Incucyte SX5 Live-cell analysis instrument (Sartorius) with images taken every 4 hours for 7 days. Media changes were performed every other day. SQOR inhibitors, FC9402 and HTS07545 were added at concentrations indicated 24 hours after cell seeding and were refreshed every other day along with regular media changes.

#### Extreme limiting dilution assay (ELDA)

Cells were seeded at 1 cells/μL in 200μL of complete neurobasal media, across the top row (row A) of an ultra-low adhesion 96-well plate. 100μL of complete neurobasal media was added to all remaining wells (rows B-H). Serial dilution was prepared by pipetting and mixing 100μL of cell suspension from row A in cell free media in row B and repeating down each column of the 96-well plate. In the end, the number of cells added per well should be A=100, B=50, C=25, D=12.5, E=6.25, F=3.125, G=1.5625, and H=0.78125. 100μL of fresh media was added on days 3 and 7 with the number of wells containing spheres counted on day 10. SQOR inhibitor pretreatment experiments were performed using cells treated for 72 hours prior to initiating the ELDA assay. Experiments testing the effect of SQOR inhibitors on sphere formation were performed by adding fresh media containing the SQOR inhibitors on days 1, 3, and 7. ELDA analysis was performed using the following bioinformatic analysis tool (https://bioinf.wehi.edu.au/software/elda/)^117^. ELDA results are shown with both the slope used for determining the goodness of fit, and bar charts representing 1 / stem cell frequency.

#### Statistical analysis

GraphPad Prism (v.11, GraphPad Software) software was used for presentation and statistical analyses. Comparisons between 2 groups were performed using unpaired two-tailed *t*-tests. Comparisons between 3 or more groups were performed using one-way ANOVAs with Tukey’s multiple comparisons test. Survival analyses were conducted using log-rank (Mantel-Cox) tests. Simple linear regressions were performed for live cell imaging experiments. Chi-square tests were used to analyze stem cell frequencies for ELDA assays.

Statistical analysis of spectral features from MetaboScape processed data were further analyzed via MetaboAnalyst 5.0 (www.metaboanalyst.ca). Zero/missing values were replaced by 1/5 of the minimum peak height over all samples, normalized by sum normalization, log 10 transformed and autoscaled prior to downstream statistical analysis. A raw p-value less than 0.05 was chosen for significance. All p-values were corrected via the Benjamini-Hochberg procedure for false discovery rate (FDR) with a threshold of 0.1. Fold change analysis was performed. Unsupervised multivariate principal component analysis (PCA) and hierarchical clustering were performed for understanding metabolite variation and expression patterns between groups. Note: statistical analysis was performed by comparing samples from male and female mice, Tumor vs Control and Non-Tumor vs Control, respectively.

## Supplementary Material

1

Supplementary Files

This is a list of supplementary files associated with this preprint. Click to download.

- OligoSequencesExcelTable.xlsx

## Figures and Tables

**Figure 1.**
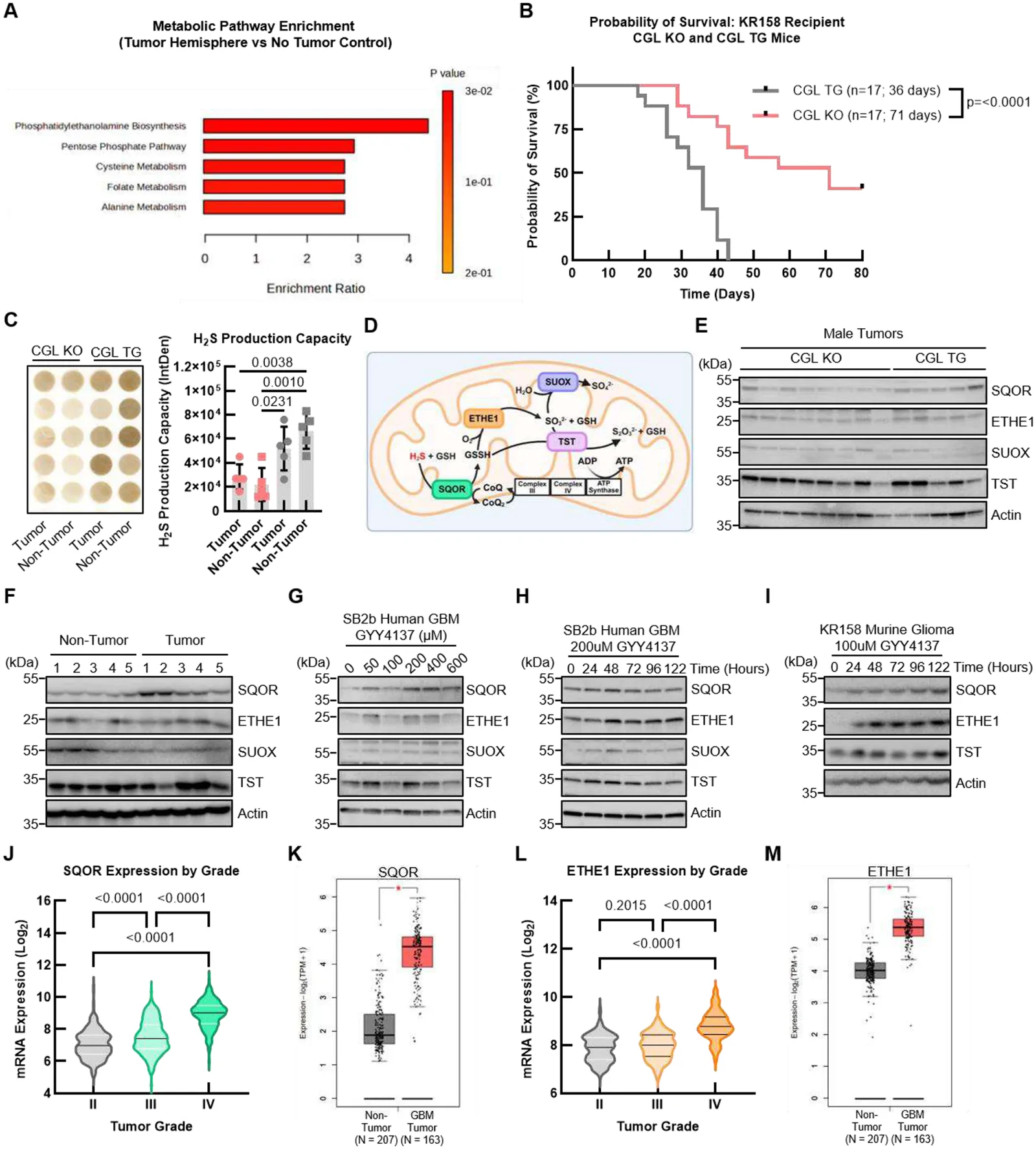
Sulfide oxidation increases in glioblastoma in response to hydrogen sulfide (H_2_S) exposure. **(A)** Metabolic pathway enrichment analysis for untargeted metabolomics of the tumor bearing hemisphere from 8-week-old C57BL/6J mice (*n*=10 per group) relative to control, non-tumor bearing brain tissue (n=5 per group) at day 32 after intracranial implantation of KR158 tumors. (**B)** Survival analysis of CGL TG (n=17 per group) and CGL KO (n=17 per group) mice after intracranial implantation of KR158 tumors. Median survival times are indicated. **(C)** Left, lead acetate assay measuring H_2_S production capacity in tissues from the tumor bearing hemisphere of CGL TG and CGL KO mice collected at endpoint. Right, quantification of lead acetate assay results measuring integrated density. **(D)** Sulfide oxidation pathway tracing the flow of sulfur from H_2_S to downstream metabolites. H_2_S is oxidized by sulfide quinone oxidoreductase (SQOR) resulting in oxidation of reduced glutathione (GSH), forming a glutathione disulfide (GSSH) with electrons being donated to coenzyme Q (CoQ) leading to the formation of reduced CoQH_2_. Persulfide Dioxygenase (ETHE1) in the presence of oxygen (O_2_) catalyzes the conversion of GSSH to GSH and sulfite (SO_3_^2-^). In the final step of the sulfide oxidation pathway, sulfite can be converted by sulfite oxidase (SUOX) into sulfate (SO_4_^2-^). Alternatively, rhodanese (TST) can convert sulfite into thiosulfate (S_2_O_3_^2-^) in the final step through the addition of a sulfur from GSSH, a process that can bypass ETHE1 all together in the presence of GSSH and sulfite. **(E)** Western blot analysis of sulfide oxidation enzyme levels with actin used as a loading control in brain tumor homogenates from male, CGL TG and CGL KO mice collected at endpoint. **(F)** Western blot analysis of sulfide oxidation enzyme levels in the matching tumor bearing and contralateral non-tumor hemispheres of 8-week-old C57BL/6J mice with an actin loading control. **(G)** Representative western blots showing sulfide oxidation enzyme levels in SB2b human glioblastoma cells stimulated with increasing concentrations of H_2_S donor molecule GYY4137 (50–600μM) for 24 hours. **(HI)** Representative western blots showing sulfide oxidation enzyme levels in **(H)** SB2b cells stimulated with 200μM GYY4137 and **(I)** KR158 mouse glioma cells stimulated with 100μM GYY4137 for increasing periods of time (24–122 hours). **(J)** Analysis of TCGA transcriptional data showing SQOR expression levels in patient glioblastoma samples based upon increasing tumor grade. **(K)** TCGA and GTEx data comparing non-tumor and tumor SQOR expression levels. **(L)** Analysis of TCGA transcriptional data showing ETHE1 expression levels in patient glioblastoma samples based upon increasing tumor grade. **(M)** TCGA and GTEx data comparing non-tumor and tumor ETHE1 expression levels. Results are representative of 3 independent experiments **(G-I)**. Data are the mean ±s.d. **(C,J-M)**. *n* indicates the number of independent animals. Statistics used: log-rank (Mantel-Cox) test **(B)**, one-way ANOVA analysis with Tukey’s multiple comparison test **(C,J,L)**, or unpaired two-tailed *t*-test **(K,M)**, only comparisons with significant p-values <0.05 were indicated.

**Figure 2.**
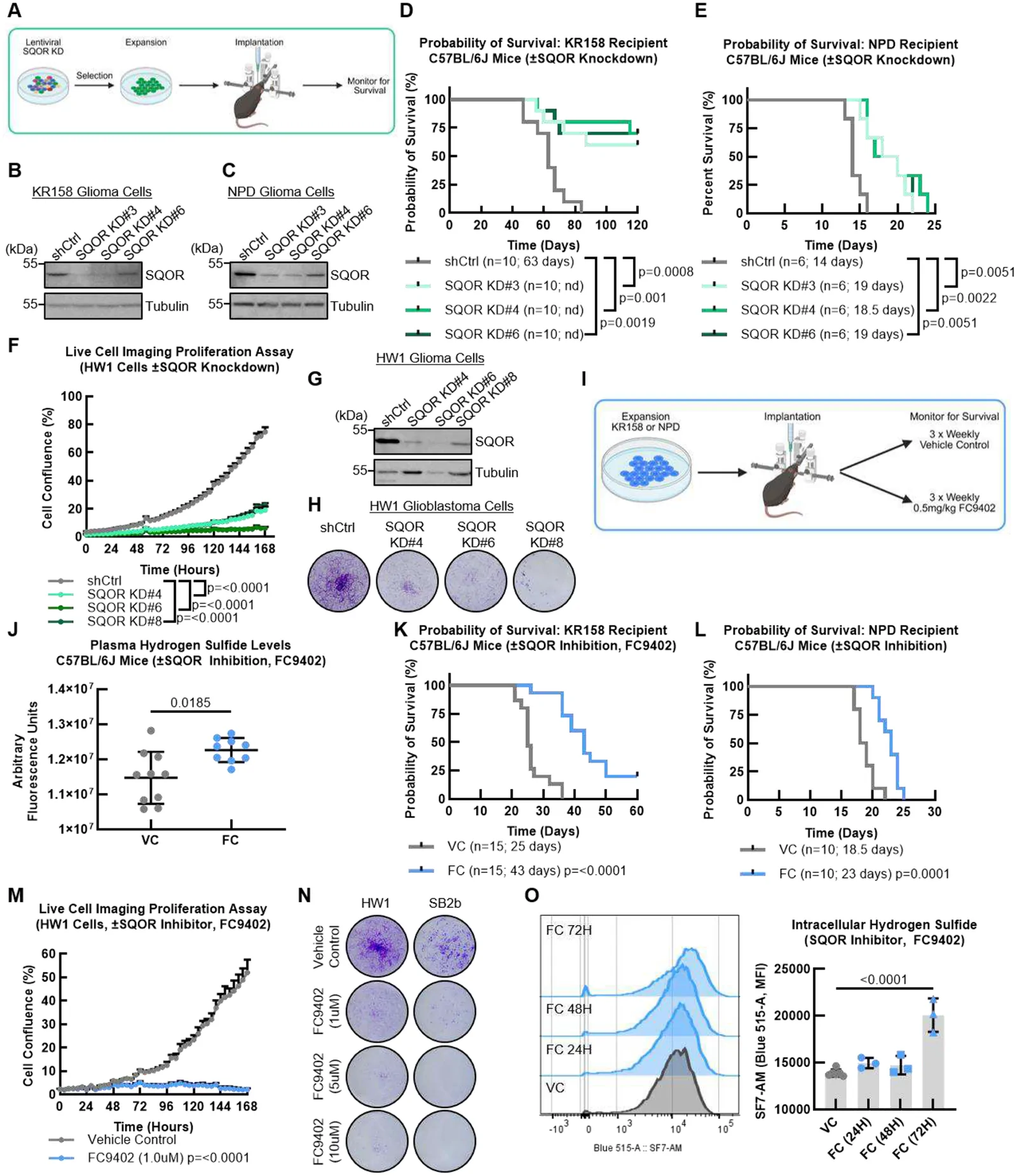
Loss of SQOR activity decreases glioblastoma growth. **(A)** Schematic diagram for generation of SQOR knockdown murine (KR158 and NPD) glioma cells, followed by intracranial implantation. **(B-C)** Representative western blot analysis confirming SQOR knockdown in murine glioma cells using **(B)** KR158 and **(C)** NPD using tubulin as a loading control. **(D-E)** Survival analysis of C57BL/6J mice after intracranial implantation with control (shCtrl) or SQOR knockdown cells generated using 3 separate shRNAs (SQOR KD#3, SQOR KD#4, and SQOR KD#6) in **(D)** KR158 tumors and **(E)** NPD tumors. **(F)** Incucyte live cell imaging analyzing cell growth as determined by cell confluency (%) in HW1 cells following SQOR knockdown. **(G)** Representative western blot analysis confirming SQOR knockdown in human glioblastoma HW1 cells. **(H)** Colony formation assay in HW1 cells following SQOR knockdown. **(I)** Schematic depicting experimental design for *in vivo* SQOR inhibitor experiments where mice were treated 3x weekly with a vehicle control or 0.5mg/kg FC9402 via intraperitoneal injection, 7 days after intracranial implantation. **(J)** Measurement of plasma H_2_S levels with the fluorescent SF7-AM probe from endpoint, tumor bearing C57BL/6J mice treated with the SQOR inhibitor. **(K-L)** Survival analysis of vehicle control and FC9402 treated C57BL/6J mice that were intracranially implanted with **(K)** KR158 and **(L)** NPD cells. **(M)** Incucyte live cell imaging of HW1 cells treated with the vehicle control of 1μM FC9402 showing % change in cell confluency as a measure of cell growth. **(N)** Colony formation assay in HW1 and SB2b cells that were treated with increasing concentrations (1–10μM) of the SQOR inhibitor, FC9402. **(O)** Left, histogram of Intracellular H_2_S measured by flow cytometry using the fluorescent probe, SF7-AM. Right, median fluorescence intensity (MFI) of H_2_S measured in HW1 cells treated with the vehicle control or 1μM FC9402 for increasing periods of time, 24, 48, and 72 hours. Results are representative of 3 independent experiments **(B,C,F-H,N-O)**. Data are the mean ±s.d. **(F,J,M,O)**. *n* indicates the number of independent animals. Statistics used: log-rank (Mantel-Cox) test **(D,E,K,L)**, simple linear regression **(F,M)**, unpaired two-tailed *t*-test **(J)**, or one-way ANOVA analysis with Tukey’s multiple comparison test **(O)**, only comparisons with significant p-values <0.05 were indicated.

**Figure 3.**
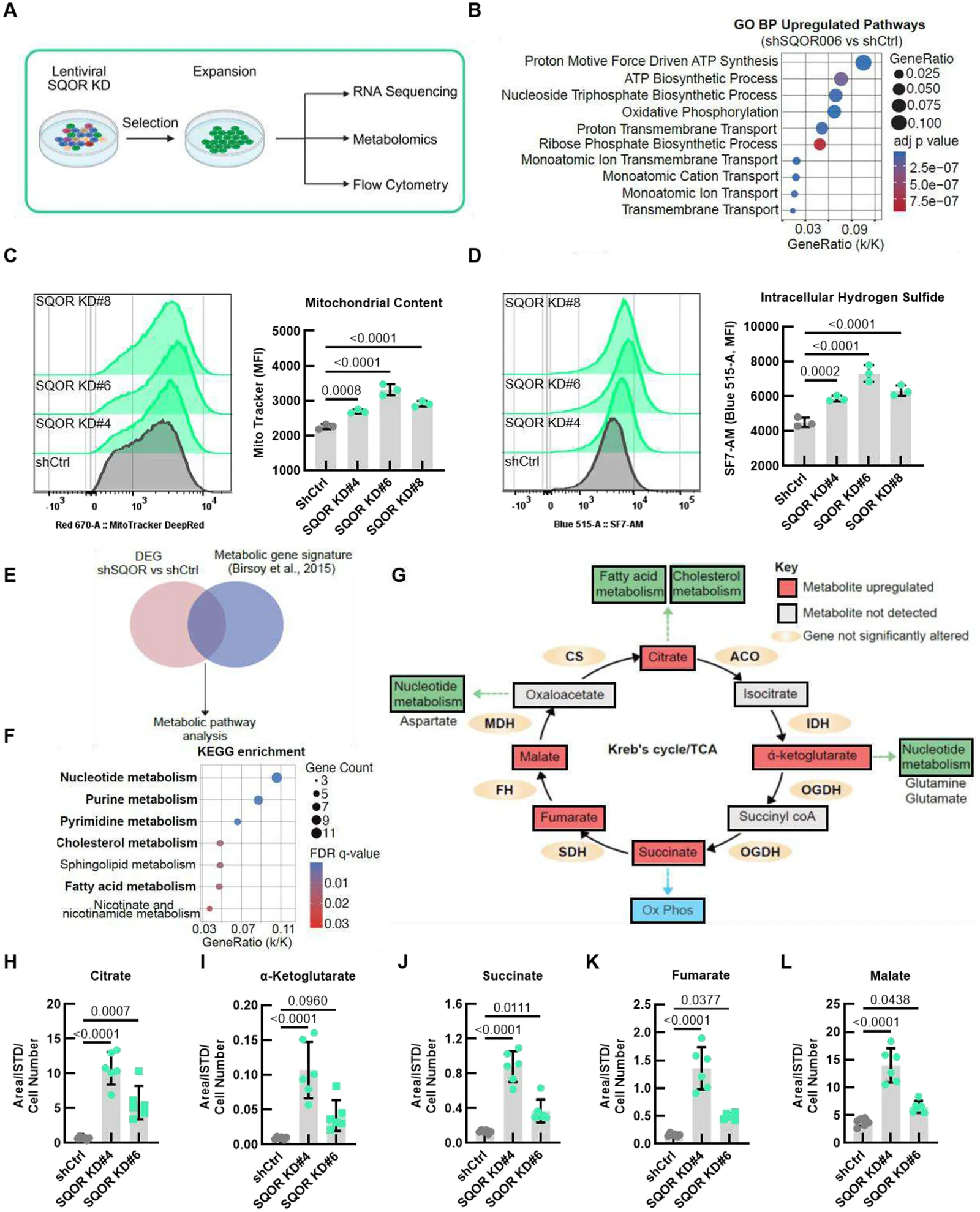
SQOR knockdown results in impaired mitochondrial function and accumulation of TCA cycle metabolic intermediates. **(A)** Schematic illustrating the generation of SQOR knockdown human HW1 glioblastoma cells for subsequent RNA sequencing, untargeted metabolomics, and flow cytometry to measure intracellular H_2_S and mitochondrial content. **(B)** GO term enrichment analysis based upon differentially expressed genes (DEGs), upregulated in SQOR KD#6 cells relative to the shCtrl cells. *n*=3 per group. **(C)** Left, histogram of mitochondrial content measured by flow cytometry using the mitotracker deep red fluorescent probe in shCtrl and SQOR knockdown HW1 cells. Right, MFI of mitotracker deep red measured in HW1 cells. **(D)** Left, histogram of Intracellular H_2_S measured by flow cytometry using the fluorescent probe, SF7-AM. Right, median fluorescence intensity (MFI) of H_2_S measured in HW1 cells following knockdown of SQOR. **(E)** Evaluation of DEGs observed following SQOR knockdown and the overlap with published metabolic gene sets (Birsoy et al., 2015) to identify metabolic pathways enriched in the absence of sulfide oxidation. **(F)** KEGG enrichment of metabolic pathways that were found to be upregulated in SQOR deficient cells. **(G)** Combined results of RNA sequencing and metabolomics are shown in the schematic depicting TCA cycle related genes, upregulated metabolites, undetected metabolites, and linked nucleotide or lipid metabolic pathways as indicated. **(H-L)** TCA cycle metabolites **(H)** citrate, **(I)** α-ketoglutarate, **(J)** succinate, **(K)** fumarate, and **(L)** malate detected using untargeted metabolomics with results normalized to D4-Taurine and cell number. *n*=6 per group. Results are representative of 3 independent experiments **(C,D)**. Data are the mean ±s.d. **(C,D,H-L)**. *n* indicates the number of replicates. Statistics used: one-way ANOVA analysis with Tukey’s multiple comparison test **(C,D,H-L)**, only comparisons with significant p-values <0.05 were indicated.

**Figure 4.**
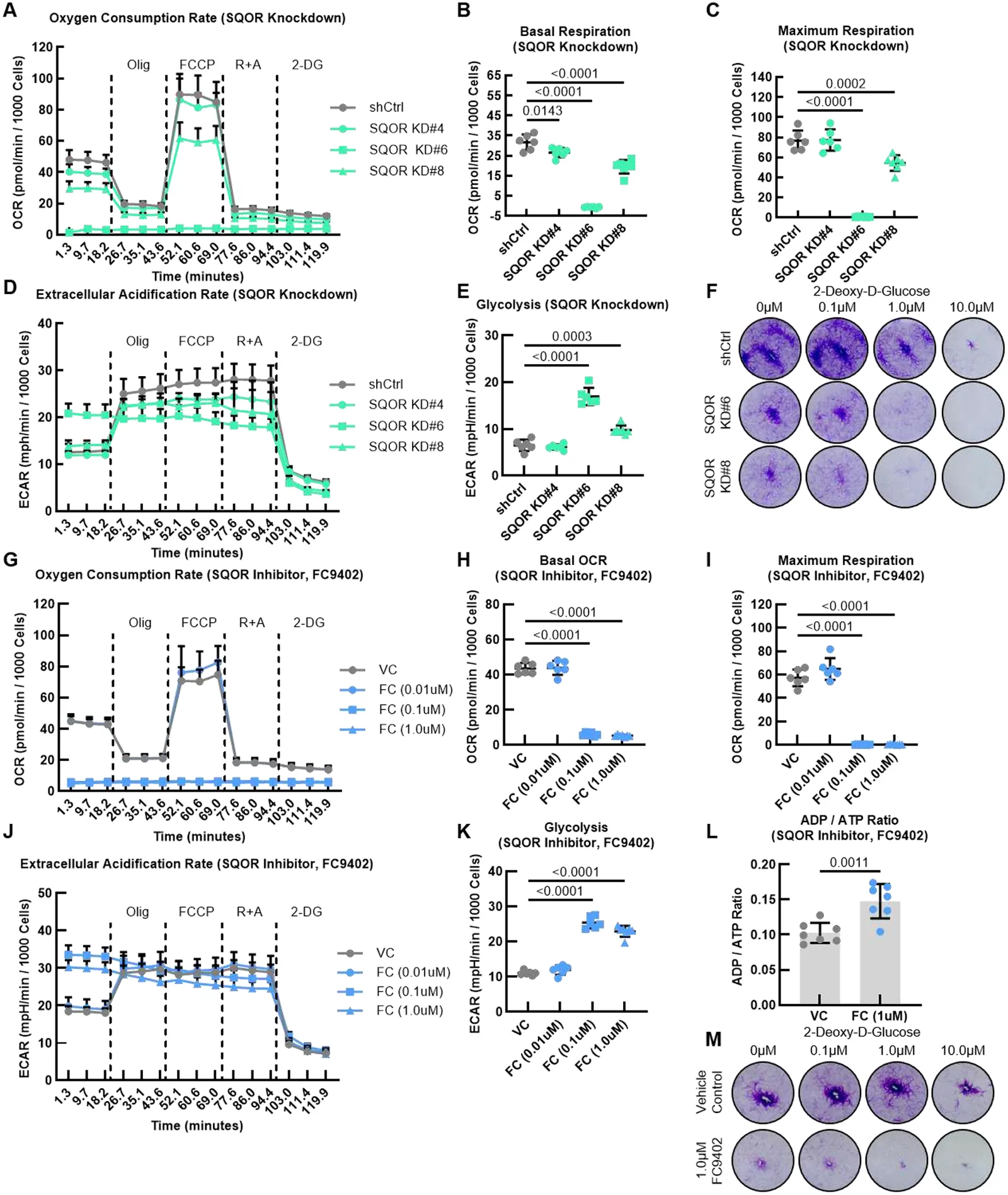
Decreasing sulfide oxidation capacity in glioblastoma disrupts mitochondrial function, leading to increased reliance upon glycolysis. **(A-E)** Seahorse XFe mitostress test results with SQOR knockdown. *n*=6 replicates per group. **(A)** Oxygen consumption Rate (OCR) of shCtrl and SQOR knockdown HW1 glioblastoma cells. **(B)** Basal respiration following SQOR knockdown. **(C)** Maximum respiration with SQOR knockdown. **(D)** Extracellular acidification rate (ECAR) with SQOR knockdown. **(E)** Glycolysis determined from ECAR measured after SQOR was knocked down. **(F)** Colony formation assay of shCtrl and SQOR knockdown HW1 glioblastoma cells with increasing concentrations (0.1–10μM) of 2-Deoxy-D-Glucose to inhibit glycolysis. **(G-L)** Seahorse XFe mitostress test results of HW1 glioblastoma cells treated with the SQOR inhibitor, FC9402 at increasing concentrations (0.01–1μM). *n*=6 replicates per group. **(G)** OCR of FC9402 treated HW1 glioblastoma cells, **(H)** basal respiration, **(I)** maximum respiration, **(J)** ECAR, and **(K)** glycolysis determined from ECAR measured in cells treated with the SQOR inhibitor, FC9402. **(L)** ADP and ATP measurements in HW1 cells treated with vehicle control (VC) or 1μM FC9402 (FC). Results are represented as the ratio of ADP/ATP. *n*=6 replicates per group. **(M)** Colony formation assay of vehicle control and FC9402 (1μM) treated HW1 glioblastoma cells with increasing concentrations (0.1–10μM) of 2-Deoxy-D-Glucose to inhibit glycolysis. Results are representative of 3 independent experiments **(A-M)**. Data are the mean ±s.d. **(B,C,E,H,I,K,L)**. *n* indicates the number of replicates. Statistics used: one-way ANOVA analysis with Tukey’s multiple comparison test **(B,C,E,H,I,K)** or unpaired two-tailed *t*-test **(L)**, only comparisons with significant p-values <0.05 were indicated.

**Figure 5.**
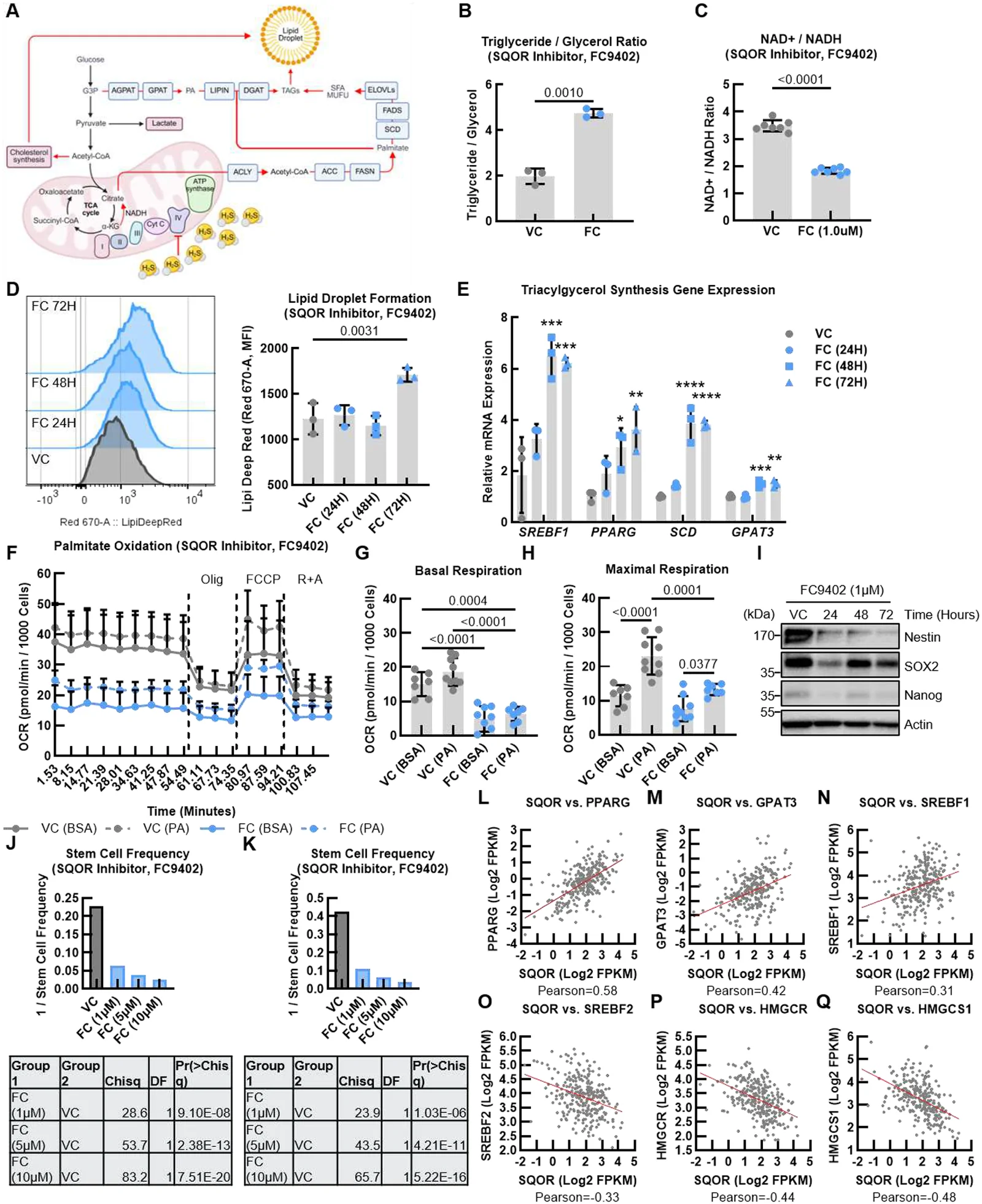
Inhibition of sulfide oxidation in glioblastoma induces lipid accumulation and decreases cancer stem cell abundance. **(A)** Schematic illustrating the flow of carbons between glycolysis, the TCA cycle, and lipid synthesis including triacyl glycerides (TAGs) and cholesterol. Inhibition of SQOR and the resulting accumulation of both H_2_S and NADH, inhibits oxidative phosphorylation leading to an increase in the synthesis of lipids stored in lipid droplets. **(B)** Measurement of triglyceride and free glycerol levels, in HW1 cells treated with the SQOR inhibitor, FC9402 (1μM) for 24 hours. Results are represented as the ratio of triglycerides/glycerol. **(C)** Measurement of NAD+ and NADH levels, in HW1 cells treated with the SQOR inhibitor, FC9402 (1μM) for 24 hours. Results are represented as the ratio of NAD+/NADH. **(D)** Left, histogram of lipid droplets measured by flow cytometry using the fluorescent LipiDeep Red probe. Right, median fluorescence intensity (MFI) of LipiDeep Red signal measured in HW1 cells treated with the vehicle control or 1μM FC9402 for increasing periods of time, 24, 48, and 72 hours. **(E)** Evaluation of triacyl glyceride synthesis related genes, *SREBF1*, *PPARG*, *SCD*, and *GPAT3*. HW1 cells were treated for 24, 48, and 72 hours with FC9402 (1μM), and the results were normalized to vehicle controls treated samples. **(F-H)** Seahorse XFe palmitate oxidation stress test results from HW1 cells treated with the SQOR inhibitor, FC9402 (1μM) in the presence of BSA conjugate control or BSA conjugated palmitic acid. **(F)** OCR of FC9402 treated HW1 cells, **(G)** basal respiration, **(H)** maximum respiration. **(I)** Western blotting was performed to evaluate cancer stem cell markers Nestin, SOX2, and Nanog with tubulin used as a loading control in FC9402 (1μM) treated HW1 cells. **(J)** Top, extreme limiting dilution assay to determine the effect of FC9402 pretreatment on L2 human glioblastoma cells with results calculated and plotted as 1/stem cell frequency. Bottom, results of chi-square test. **(K)** Top, extreme limiting dilution assay to determine the effect of FC9402 treatment on L2 human glioblastoma cells with results calculated and plotted as 1/stem cell frequency. Bottom, results of chi-square test. **(L-Q)** Analysis of TCGA data examining co-expression of SQOR and lipid metabolism related genes. **(L)** SQOR and PPARG, **(M)** GPAT3, **(N)** SREBF1, **(O)** SREBF2, **(P)** HMGCR, **(Q)** HMGCS1. Results are representative of 3 independent experiments **(B-K)**. Data are the mean ±s.d. **(B-E,G,H)**. *n*=3–12 replicates per group for each experiment. Statistics used: unpaired two-tailed *t*-test **(B,C)**, one-way ANOVA analysis with Tukey’s multiple comparison test **(D,E,G,H)**, chi-square test **(J,K)**, Pearson’s correlation analysis **(L-Q)**, only comparisons with significant p-values <0.05 were indicated.

**Figure 6.**
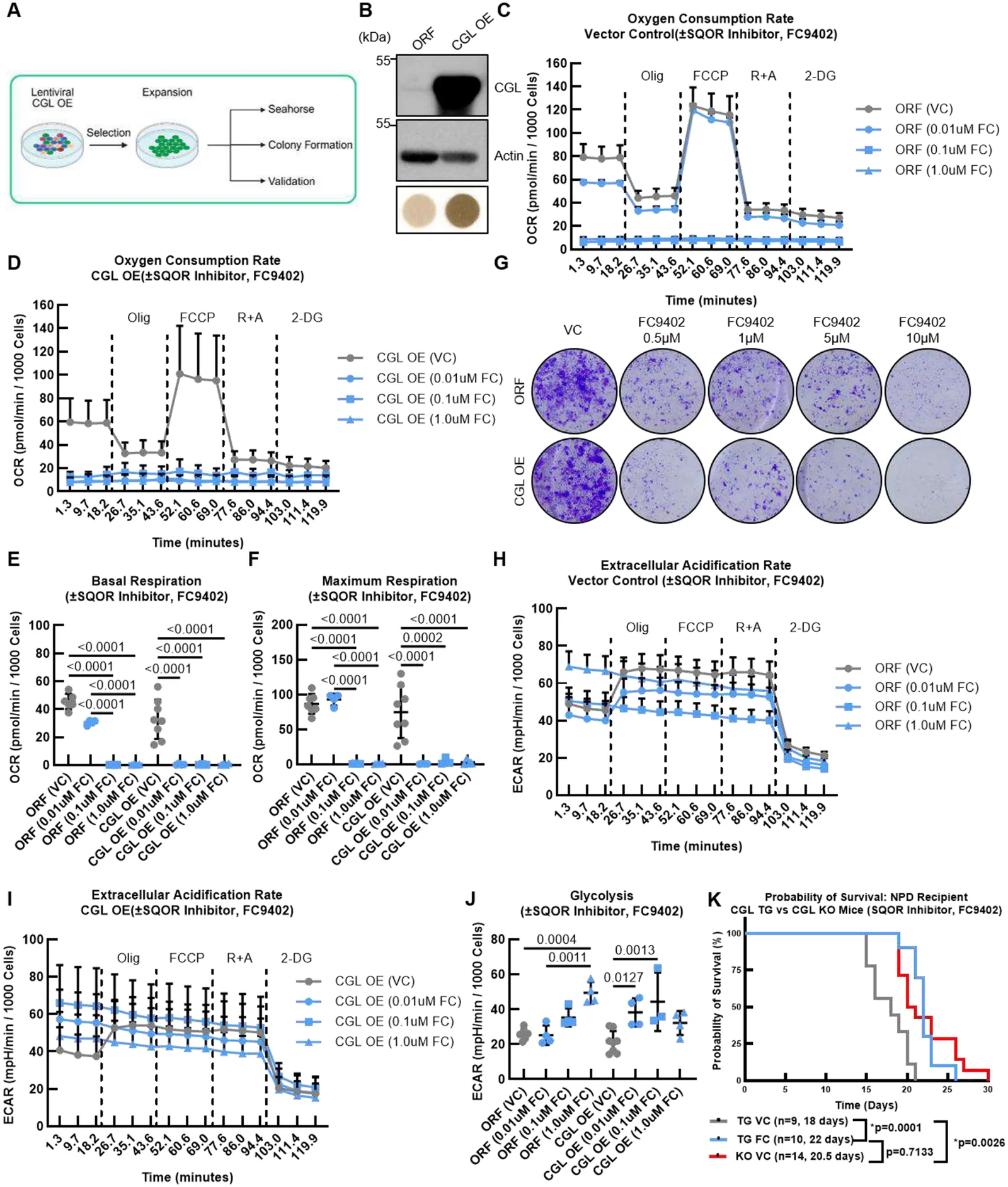
Targeting SQOR under conditions where H_2_S levels are increased sensitizes glioblastoma cells to inhibition of sulfide oxidation. **(A)** Schematic depicting the experimental setup for CGL overexpression to be combined with SQOR inhibition using FC9402. **(B)** Western blot and lead acetate analysis to confirm CGL overexpression (CGL OE) and increased H_2_S production capacity. **(C-F and H-J)** Seahorse XFe mitostress test results *n*=3–7 replicates per group of open reading frame (ORF) controls and CGL overexpressing (CGL OE) HW1 cells. ORF and CGL OE cells were treated with either vehicle control (VC) or increasing concentrations of FC9402 (0.01–1μM). Vehicle controls were the same as plotted in supplementary figure 6. **(C)** Oxygen consumption Rate (OCR) of ORF cells treated with FC9402. **(D)** Oxygen consumption rate of CGL OE cells treated with FC9402. **(E)** Basal respiration of ORF and CGL OE cells treated with FC9402. **(F)** Maximum respiration of ORF and CGL OE cells treated with HTS07545. **(G)** Colony formation assay of ORF and CGL OE SB2b human glioblastoma cells treated with increasing concentrations (0.5–10μM) of FC9402. **(H)** ECAR of FC9402 treated, ORF HW1 cells. **(I)** ECAR of FC9402 treated CGL OE cells. **(J)** Glycolysis determined from ECAR measured in ORF and CGL OE HW1 cells treated with the SQOR inhibitor, FC9402. (**K**) Survival analysis of vehicle control and FC9402 treated CGL TG or CGL KO mice that were intracranially implanted with NPD cells. Results are representative of 3 independent experiments **(B-J)**. Data are the mean ±s.d. **(E,F,J)**. *n* indicates the number of replicates per group. Statistics used: one-way ANOVA analysis with Tukey’s multiple comparison test **(E,F,J)**, only comparisons with significant p-values <0.05 were indicated.

**Figure 7.**
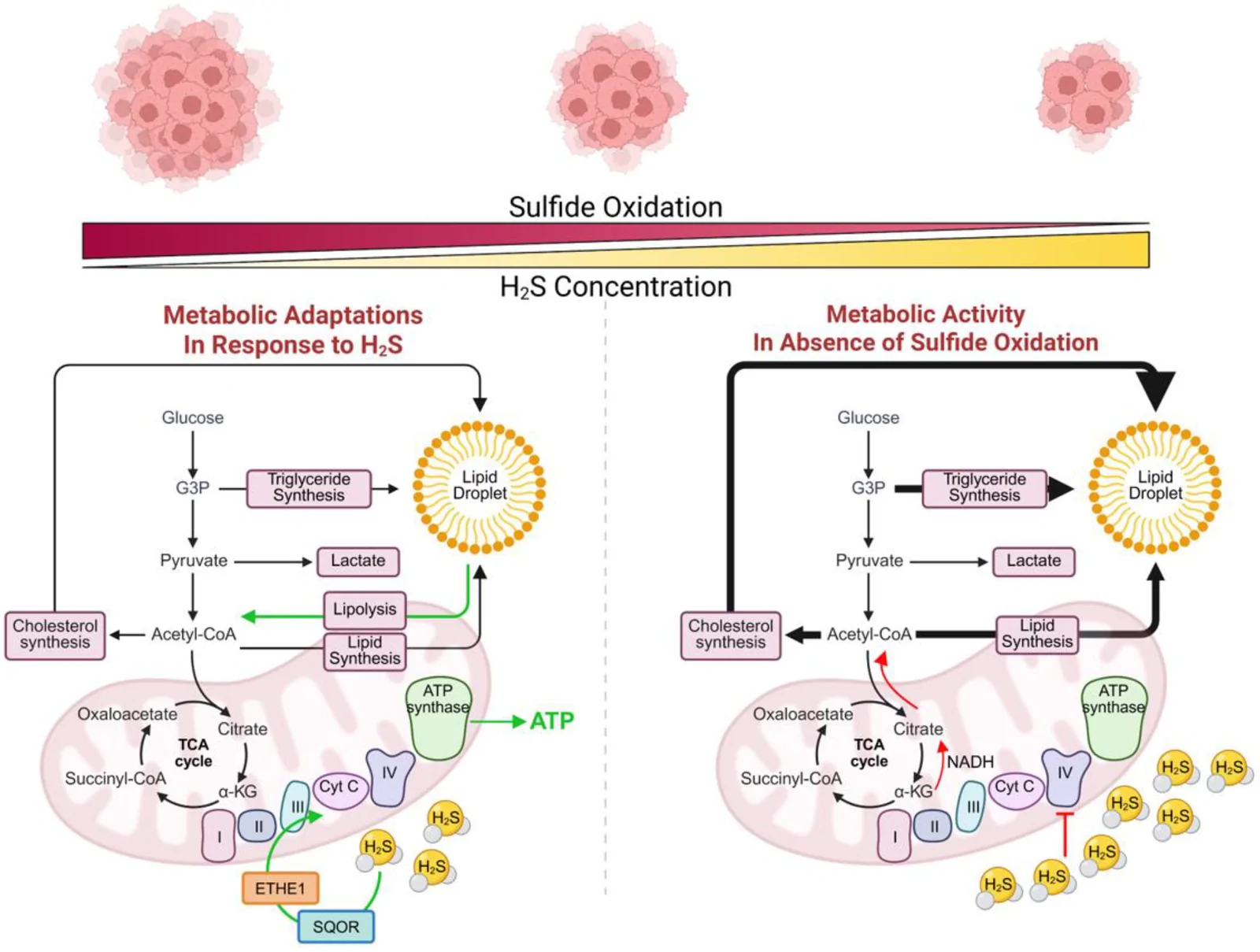
Model figure for leveraging H_2_S as a GBM tumor-suppressive molecule via SQOR activity. Left: Sulfide oxidation is increased in glioblastoma in response to endogenously and exogenously produced H_2_S. Oxidation of H_2_S maintains normal mitochondrial function, a process that enables oxidative phosphorylation to offset energetic demands, including the mobilization of lipids under periods of low glucose availability. Right: In the absence of sulfide oxidation, H_2_S levels rise, leading to inhibition of mitochondrial respiration, accumulation of TCA cycle metabolic intermediates, and increased synthesis and storage of lipids in lipid droplets that are associated with decreased tumor growth and disease progression in glioblastoma.

**Table:** Key resources table

REAGENT or RESOURCE	SOURCE	IDENTIFIER
Antibodies		
Rabbit Polyclonal anti-SQRDL	Thermo Fisher Scientific	Cat# PA5-53549; RRID:AB_2647905
Rabbit Polyclonal anti-ETHE1	Thermo Fisher Scientific	Cat# PA5-56040; RRID:AB_2641135
Mouse Monoclonal anti-TST	Proteintech	Cat# 66018-1-1g; RRID:AB_11045660
Rabbit Monoclonal anti-SUOX	Abcam	Cat# Ab129094; RRID:AB_11143396
Rabbit Polyclonal anti-SUOX	Proteintech	Cat# 15075-1-AP; RRID:AB_2198558
Mouse Monoclonal anti-CGL	Abnova	Cat# H00001491-M01; RRID:AB_1574152
Rabbit Monolclonal anti-CBS	Cell Signaling Technology	Cat# 14782S; RRID:AB2798609
Rabbit Polyclonal anti-MPST	Thermo Fisher Scientific	Cat# PA5-51548; RRID:AB_2644090
Mouse Monoclonal anti-Nestin	Thermo Fisher Scientific	Cat# MA1-110; RRID:AB_2536821
Rabbit Polyclonal anti-SOX2	Cell Signaling Technology	Cat# 2748S; RRID:AB_823640
Rabbit Monoclonal anti-Nanog	Cell Signaling Technology	Cat# 8822S; RRID:AB_11217637
Rabbit Polyclonal anti-Phospho-ATP-Citrate Lyase (Ser455)	Cell Signaling Technology	Cat# 4331T; RRID:AB_2257987
Rabbit Polyclonal anti-ATP-Citrate Lyase	Cell Signaling Technology	Cat# 4332T; RRID:AB_2223744
Rabbit Monoclonal anti-Lipin 1	Cell Signaling Technology	Cat# 14906T; RRID:AB_2798644
Rabbit Monoclonal anti-ACSL1	Cell Signaling Technology	Cat# 9189T; RRID:AB_10891616
Rabbit Monoclonal anti-ACeCS1	Cell Signaling Technology	Cat#3658T; RRID:AB_2222710
Rabbit Polyclonal anti-alpha Tubulin	Abcam	Cat# Ab4074; RRID:AB_2288001
Rabbit Polyclonal anti-beta Actin	Abcam	Cat# Ab8227; RRID:AB_2305186
OxPhos Human WB Antibody Cocktail	Thermo Fisher Scientific	Cat# 45-8199; RRID:AB_2533836
Bacterial and virus strains		
		
		
Biological samples		
Patient Derived Xenograft: HW1	Stringer et al.^102^	RRID:CVCL_VS45
Patient Derived Xenograft: WK1	Stringer et al.^102^	RRID:CVCL_VS54
Patient Derived Xenograft: MMK1	Stringer et al.^102^	RRID:CVCL_VS47
Patient Derived Xenograft: PB1	Stringer et al.^102^	RRID:CVCL_VS49
Patient Derived Xenograft: SB2b	Stringer et al.^102^	RRID:CVCL_VS52
Patient Derived Xenograft: RN1	Stringer et al.^102^	RRID:CVCL_VS51
Patient Derived Xenograft: 23	Silver et al.^6^	
Patient Derived Xenograft: L0	Deleyrolle et al.^105^	
Patient Derived Xenograft: L1	Deleyrolle et al.^105^	
Patient Derived Xenograft: L2	Deleyrolle et al.^105^	
Patient Derived Xenograft: T4121	Bao et al.^108^	
Patient Derived Xenograft: T3832	Zhizhong et al.^107^	
Patient Derived Xenograft: T3691	Zhizhong et al.^107^	
Patient Derived Xenograft: DI318	Mitchell et al.^109^	
Chemicals, peptides, and recombinant proteins		
Accutase - Enzyme Cell Detachment Medium	Thermo Fisher Scientific	Cat# 00-4555-56
DMEM/F12	Cleveland Clinic Media Core	Cat# 13-500
Neurobasal Medium, Minus Phenol Red	Thermo Fisher Scientific	Cat# 12348017
D-PBS	Cleveland Clinic Media Core	Cat# 121-500
HBSS	Cleveland Clinic Media Core	Cat# 161-500
Pen/Strep; 10,000 u/mL	Cleveland Clinic Media Core	Cat# 725-100
L-Glutamine	Cleveland Clinic Media Core	Cat# 523-100
Sodium Pyruvate	Thermo Fisher Scientific	Cat# 11360070
N-2 Supplement (100x)	Thermo Fisher Scientific	Cat# 17502048
B-27 Supplement (50x)	Thermo Fisher Scientific	Cat# 17504044
Recombinant Human EGF	R&D Systems	Cat# 236-EG
Recombinant Human FGF	R&D Systems	Cat# BT-FGFB
Geltrex LDEV-Free Reduced Growth Factor Basement Membrane Matrix	Fisher Scientific	Cat# A1413202
RIPA Lysis and Extraction Buffer	Thermo Fisher Scientific	Cat# 89900
2-Mercaptoethanol	Sigma-Aldrich	Cat# M6250-100ML
2x Laemmli Sample Buffer	BioRad	Cat# 1610737EDU
5x Passive Lysis Buffer	Fisher Scientific	Cat# 50-196-5009
Halt Protease Inhibitor Cocktail (100x)	Thermo Fisher Scientific	Cat# 78438
Halt Phosphatase Inhibitor Cocktail Single-Use Cocktail	Thermo Fisher Scientific	Cat# 78428
PEG400	MedChemExpress	Cat# HY-Y0873A
Tween-80	MedChemExpress	Cat# HY-Y1891
Sodium Chloride, 0.1N Standardized Solution	Thermo Fisher Scientific	Cat# 035616.AP
FC9402	MedChemExpress	Cat# HY141552
HTS07545	MedChemExpress	Cat# HY-144439
GYY4137	Cayman Chemical	Cat# 13345
SF7-AM	Cayman Chemical	Cat# 14623
Dimethyl Sulfoxide	Fisher Scientific	Cat# BP231-100
Lipi-Deep Red Lipid Droplet Staining	Dojindo	Cat# LD04-10
LIVE/DEAD Fixable Blue Dead Cell Stain Kit	Thermo Fisher Scientific	Cat# L23105
MitoTracker Deep Red FM Dye	Thermo Fisher Scientific	Cat# M46753
Propidium Iodide	Thermo Fisher Scientific	Cat# R37169
Bovine Serum Albumin	Fisher Scientific	Cat# BP1600-100
PageRuler Plus Prestained Protein Ladder	Thermo Fisher Scientific	Cat# 26620
10x Tris/Glycine/SDS Running Buffer	BioRad	Cat# 1610772
10x Tris/Glycine Transfer Buffer	BioRad	Cat# 1610771
TRIS-Buffered Saline (TBS, 10x) pH 7.4	Thermo Scientific	Cat# J60764.K2
Blotto, non-fat dry milk	Santa Cruz Biotechnology	Cat# sc-2324
Restore PLUS Western Blot Stripping Buffer	Thermo Scientific	Cat# 46430
Fast SYBR Green Master Mix	Applied Biosystems	Cat# 4385612
Verso cDNA Synthesis Kit	Thermo Fisher Scientific	Cat# AB1453B
2-Deoxy-D-Glucose	Millipore Sigma	Cat# D8375-5G
Oligomycin	Millipore Sigma	Cat# O4876-5MG
FCCP	Millipore Sigma	Cat# C2920-10MG
Rotenone	Millipore Sigma	Cat# R8875-1G
Antimycin A	Millipore Sigma	Cat# A8674-25MG
L-Carnitine	Cayman Chemical	Cat# 21489
Seahorse XF 1.0M glucose solution, 50mL	Agilent	Cat# 103577-100
Seahorse XF 200mM glutamine solution, 50mL	Agilent	Cat# 103579-100
Seahorse XF 100mM pyruvate solution, 50mL	Agilent	Cat# 103578-100
Seahorse XF DMEM medium, pH 7.4, 500mL	Agilent	Cat# 103575-100
BSA Control for BSA-Fatty Acid Complexes	Cayman Chemical	Cat# 29556
BSA-Palmitate Saturated Fatty Acid Complex	Cayman Chemical	Cat# 29558
Hoechst 33342, trihydrochloride trihydrate	Invitrogen	Cat# H3570
L-Cysteine	Millipore Sigma	Cat# 168149-2.5G
Pyridoxal 5’-phosphate hydrate	Millipore Sigma	Cat# P9255-1G
Lead Acetate, Trihydrate	Fisher Scientific	Cat# LC160801
Methanol	Thermo Fisher Scientific	Cat# 325740025
Crystal Violet	Thermo Fisher Scientific	Cat# 548-62-9
Puromycin Dihydrochloride	Thermo Fisher Scientific	Cat# A1113802
Geneticin	Thermo Fisher Scientific	Cat# 10131035
Critical commercial assays		
Seahorse FluxPaks	Agilent	Cat# 103792-100
ADP/ATP Ratio Assay	Dojindo	Cat# A552-10
NAD/NADH-Glo Assay	Promega	Cat# G9071
Lactate-Glo Assay	Promega	Cat# J5021
Pyruvate-Glo Assay	Promega	Cat# J4051
Triglyceride-Glo Assay	Promega	Cat# J3160
Glutamine/Glutamate-Glo Assay	Promega	Cat# J8021
Free Fatty Acid Assay Kit	Abcam	Cat# Ab65341
SuperSignal West Pico PLUS Chemiluminescent Substrate	Thermo Fisher Scientific	Cat# 34580
Bio-Rad Protein Assay Kit 1	BioRad	Cat# 5000001
Deposited data		
Untargeted Metabolomics Data	This paper	Metabolomics Workbench: datatrack_id:7895 study_id:ST005168 The DOI for this project(PR003335) is: https://doi.org/http://dx.doi.org/10.21228/M8M587
Bulk RNA Sequencing Data	This paper	NCBI GEO ID: GSE346875
Steady State Metabolomics Data	This paper	Pending Consortium Finalization
		
Experimental models: Cell lines		
KR158	Reilly et al.^72^	N/A
CT-2A	Seyfried et al.^111^	RRID:CVCL_ZJ44
SB28	Wiesner et al.^112^	N/A
GL261	Ausman et al.^113^	RRID:CVCL_Y003
NPD	Comba et al.^73^	N/A
CPA	Alghamri et al.^114^	N/A
OL61	Kamran et al.^115^	N/A
mGBM6	N/A	N/A
fGBM7	N/A	N/A
Experimental models: Organisms/strains		
Mouse: C57BL/6J	The Jackson Laboratory	JAX:00064; RRID:JAX:000664
Mouse: CGL Knockout (CGL KO)	Hine et al.^67^	N/A
Mouse: CGL WT/Transgenic (CGL TG) Overexpression	Trocha el al.^69^	N/A
		
Oligonucleotides		
Primers, see **Table S1**	This paper	N/A
		
Recombinant DNA		
MISSION^®^ pLKO.1-puro Non-Target shRNA Control Transduction Particles	Sigma-Aldrich	SHC016H-1EA
MISSION^®^ pLKO.1-puro shRNA for Human SQOR KD#4	Sigma-Aldrich	TRCN0000039004
MISSION^®^ pLKO.1-puro shRNA for Human SQOR KD#6	Sigma-Aldrich	TRCN0000039006
MISSION^®^ pLKO.1-puro shRNA for Human SQOR KD#8	Sigma-Aldrich	TRCN0000039008
MISSION^®^ pLKO.1-puro shRNA for Murine SQOR KD#3	Sigma-Aldrich	TRCN0000042373
MISSION^®^ pLKO.1-puro shRNA for Murine SQOR KD#4	Sigma-Aldrich	TRCN0000042374
MISSION^®^ pLKO.1-puro shRNA for Murine SQOR KD#6	Sigma-Aldrich	TRCN0000042376
pLV[Exp]-Puro-EF1A>ORF_Stuffer	VectorBuilder	VB010000-9667qqs
pLV[Exp]-Neo-EF1A>hCTH[NM_001902.6]	VectorBuilder	VB260108-1269bkn
Software and algorithms		
GraphPad Prism (v.11)	GraphPad Software	N/A
MetaboScape 2025b	Bruker	N/A
MetaboAnalyst 5.0		https://www.metaboanalyst.ca/
Fiji	ImageJ	N/A
Incucyte Software for Live-Cell Analysis	Sartorius	N/A
Seahorse Wave	Agilent Technologies	N/A
Agilent Seahorse Analytics	Agilent Technologies	
SoftMax Pro	Molecular Devices	N/A
FlowJo v10.10.1	BD Biosciences	N/A
Biorender	BioRender	https://www.biorender.com/

## Highlights

- Glioblastoma exploits mitochondrial sulfide oxidation to neutralize tumor-suppressive H_2_S and sustain oxidative metabolism.
- SQOR is a metabolic gatekeeper that links sulfide detoxification to electron transport and fuels tumor fitness.
- SQOR loss unleashes H_2_S toxicity, collapses mitochondrial function, and cripples the TCA cycle, forcing glycolytic compensation.
- Targeting SQOR drives lipid accumulation, blocks glioma progression, and prolongs survival *in vivo*.
