# Supplementary material for: SQOR regulates H2S detoxification and lipid metabolism to maintain glioblastoma metabolic fitness
Source: Res Sq. 2026 Sep 28:rs.3.rs-11077727. Preprint. [Version 1] doi: 10.21203/rs.3.rs-11077727/v1 (PMC13648848; doi:10.21203/rs.3.rs-11077727/v1)
Supplement: Supplementary file 1 [file NIHPP-RS11077727V1-supplement-1.pdf]

Supplementary figures and figure legends

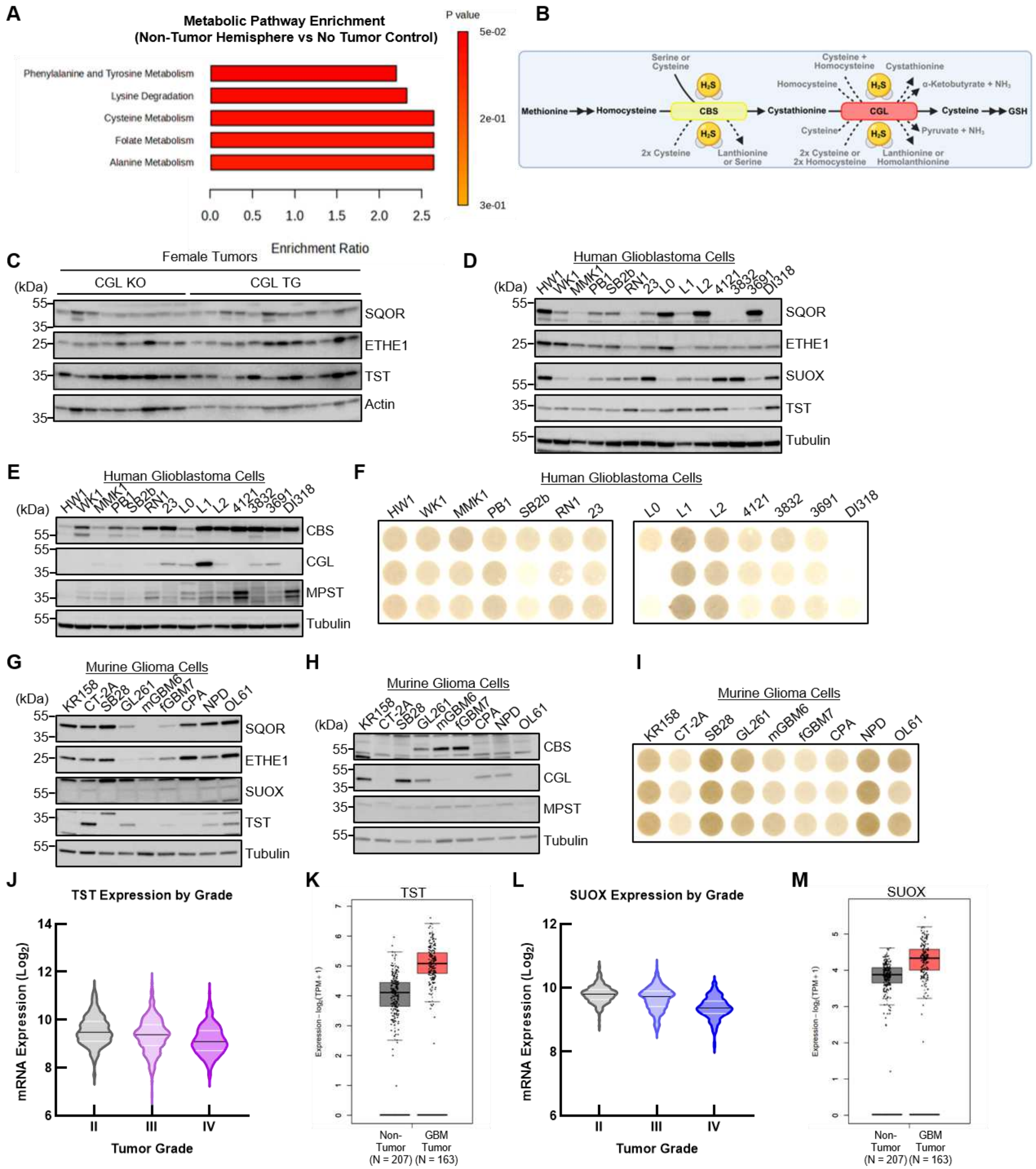

Supplementary Figure 1. Minimal basal H_2S production is observed in glioblastoma cells. (A) Metabolic pathway enrichment analysis for untargeted metabolomics of the non-tumor bearing hemisphere from 8-week-old C57BL/6J mice (n=10 per group) relative to control, non-tumor bearing brain tissue (n=5 per group) at day 32 after intracranial implantation of KR158 tumors. **(B)** Transsulfuration pathway illustrating the flow of sulfur from methionine through the production of cysteine and reduced glutathione (GSH). Enzymes upstream of the transsulfuration pathway generate homocysteine from

methionine. Homocysteine along with either serine or cysteine can be used by cystathione beta-synthase (CBS) to produce cystathionine. Alternatively, CBS can use cysteine to generate either serine or lanthionine. Enzymatic reactions capable of producing the gaseous molecule, H_2S. In the second step of the transsulfuration pathway, cystathione gamma-lyase, a promiscuous molecule can utilize cystathionine, cysteine, or homocysteine to produce cystathionine, α-ketobutyrate, ammonia (NH_3), pyruvate, lanthionine, or homolanthionine, along with H_2S. Cysteine can be utilized for various purposes including synthesis of proteins as well production of GSH. **(C)** Western blot analysis of sulfide oxidation enzyme levels with actin used as a loading control in brain tumor homogenates from female, CGL TG and CGL KO mice collected at endpoint. **(D)** Western blot analysis of sulfide oxidation enzymes across a collection of patient derived glioblastoma cells with α-tubulin used as a loading control. **(E)** Examination of H_2S producing enzyme levels at baseline in patient derived glioblastoma cells via western blotting with α-tubulin used as a loading control. **(F)** Lead acetate assay results measuring H_2S production capacity of patient derived glioblastoma cells. **(G)** Western blot analysis of sulfide oxidation enzymes across a collection of murine glioma cells with α-tubulin used as a loading control. **(H)** Examination of H_2S producing enzyme levels at baseline in patient derived glioblastoma cells via western blotting with α-tubulin used as a loading control. **(I)** Lead acetate assay results measuring H_2S production capacity of murine glioma cells. **(J)** Analysis of TCGA transcriptional data showing TST expression levels in patient, glioblastoma samples based upon increasing tumor grade. **(K)** TCGA and GTEx data comparing non-tumor and tumor TST expression levels. **(L)** Analysis of TCGA transcriptional data showing SUOX expression levels in patient, glioblastoma samples based upon increasing tumor grade. **(M)** TCGA and GTEx data comparing non-tumor and tumor SUOX expression levels. Results are representative of 3 independent experiments **(D-I)**. Data are the mean ±s.d. **(J,L)**. Statistics used: one-way ANOVA analysis with Tukey's multiple comparison test **(J,L)**, or unpaired two-tailed *t*-test **(K,M)**, only comparisons with significant p-value <0.05 were indicated.

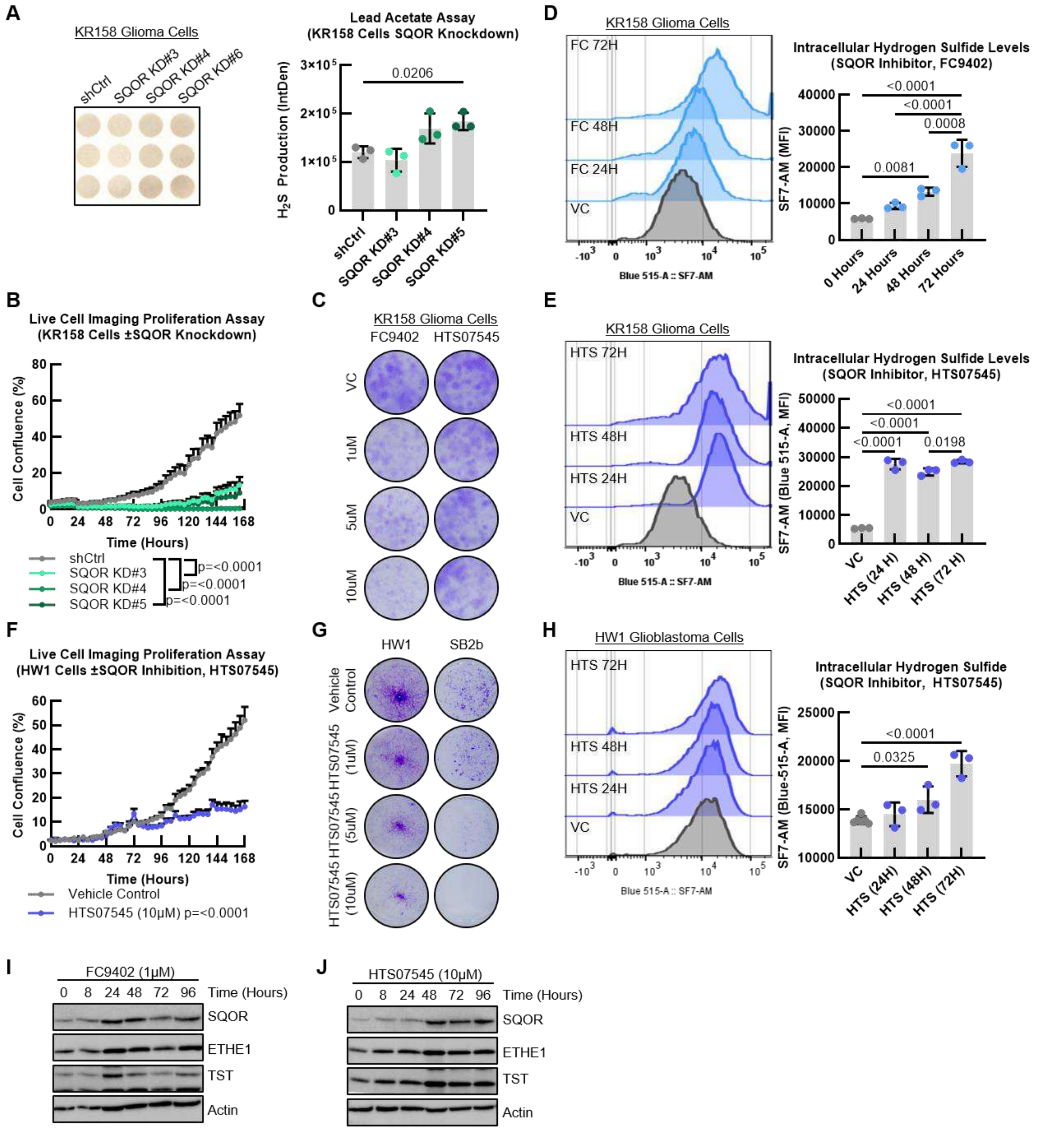

Supplementary Figure 2. Decreased capacity for sulfide oxidation results in elevated intracellular H_2S accumulation. (A) Left, Lead acetate assay measuring the effect of SQOR knockdown on H_2S production capacity. Right, quantification of lead acetate assay. **(B)** Incucyte live cell imaging measuring cell confluency (%) of shCtrl and SQOR knockdown murine glioma KR158 cells. **(C)** Colony formation assay or KR158 cells treated with increasing concentrations of SQOR inhibitors, FC9402 (1-10μM) and HTS07545 (1-10μM). **(D-E)** Flow cytometric measurement of intracellular H_2S using the fluorescent SF7-AM probe following inhibition of SQOR. **(D)** Left, histogram of SF7-AM fluorescence intensity in KR158 cells treated with 1μM FC9402 for 24, 48, and 72 hours. Right, SF7-AM MFI. **(E)** Left, histogram of SF7-AM fluorescence intensity in KR158 cells treated with 10μM HTS07545 for 24, 48, and 72 hours. Right, SF7-AM MFI. **(F)** Incucyte live cell imaging analyzing cell growth as determined by cell confluency (%) in HW1 cells following treatment with the SQOR inhibitor, HTS07545 (10μM). **(G)** Colony formation assay in HW1 and SB2b cells treated with FC9402 at increasing concentrations (1-10μM). **(H)** Left, histogram of Intracellular H_2S in HW1 cells treated for 24, 48, and 72 hours

with HTS07545 (10μM) measured by flow cytometry using the fluorescent probe, SF7-AM. Right, median fluorescence intensity (MFI) of intracellular H_2S. **(I-J)** Western blot analysis showing the effects of SQOR inhibition on sulfide oxidation pathway. **(I)** FC9402 (1μM) time course in HW1 cells treated for 8, 24, 48, 72, and 96 hours. **(J)** HTS07545 (10μM) time course in HW1 cells treated for 8, 24, 48, 72, and 96 hours. Results are representative of 3 independent experiments **(A-J)**. Data are the mean ±s.d. **(A,B,D,E,F,H)**. *n*=3 replicated per group. Statistics used: one-way ANOVA analysis with Tukey's multiple comparison test **(A,D,E,H)**, or linear regression **(B,F)**, only comparisons with significant p-values <0.05 were indicated.

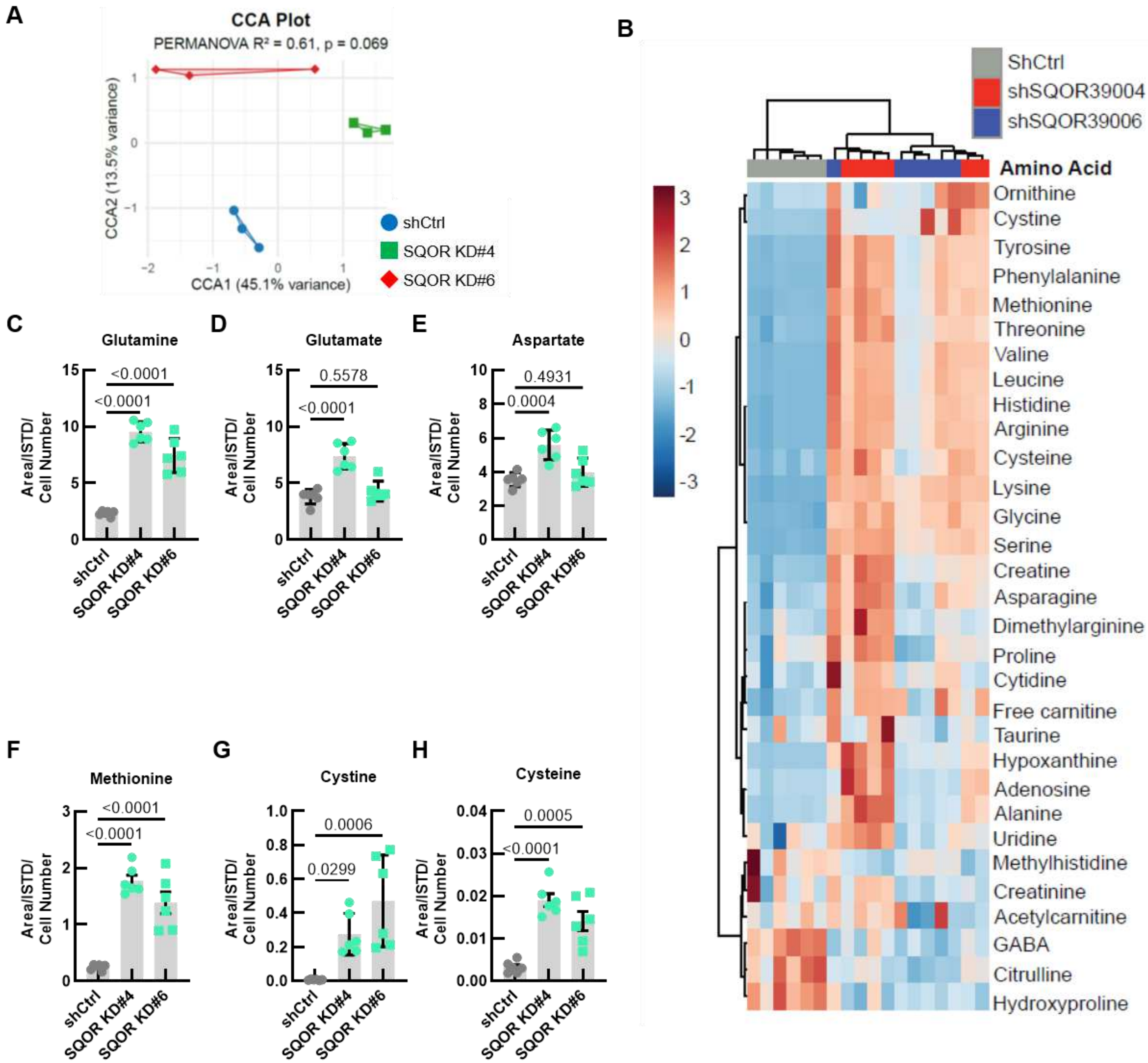

Supplementary Figure 3. Impaired mitochondrial function and accumulation of TCA cycle metabolic intermediates due to SQOR knockdown leads to increased abundance of amino acids. (A) CCA plot of RNA sequencing results from shCtrl and SQOR KD#4 and SQOR KD#6 cells. *n*=3 per group. **(B)** Heatmap of amino acids and amino acid based neurotransmitters detected in untargeted metabolomics. *n*=6 per group. **(C-E)** Amino acid related to the TCA cycle **(C)** glutamine, **(D)** glutamate, and **(E)** aspartate as well as **(F-H)** amino acids related to cysteine metabolism **(F)** methionine, **(G)** cystine, and **(H)** cysteine detected using untargeted metabolomics with results normalized to D3-Alanine and cell number. *n*=6 per group. Data are the mean ±s.d. **(C-H)**. Statistics used: one-way ANOVA analysis with Tukey's multiple comparison test **(C-H)**, only comparisons with significant p-values <0.05 were indicated.

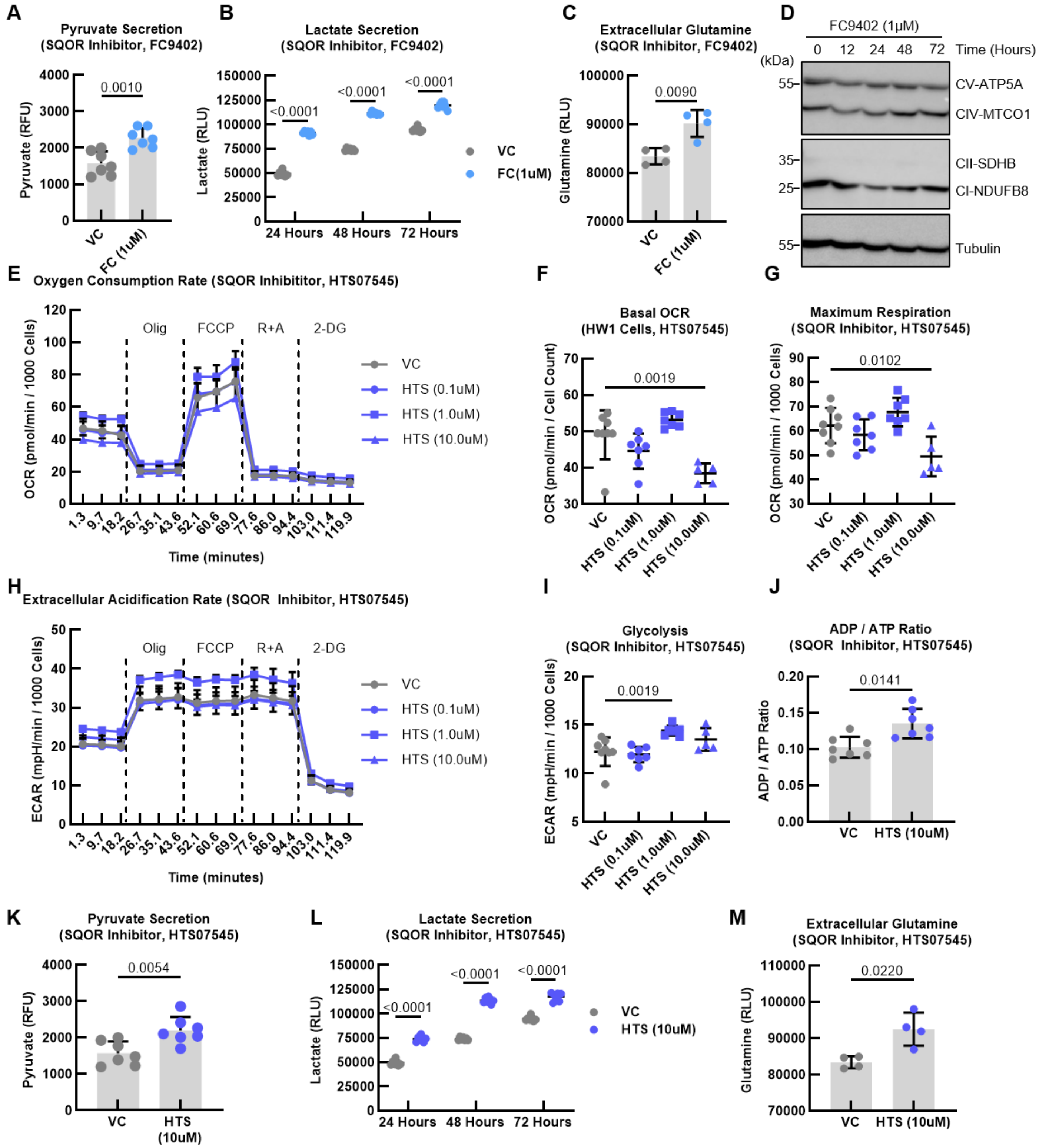

Supplementary Figure 4. Decreased sulfide oxidation capacity forces glioblastoma cells to increase reliance upon glycolysis. (A) Measurement of pyruvate secreted by human glioblastoma HW1 cells treated with the SQOR inhibitor, FC9401 (1μM) for 24 hours. **(B)** Lactate secretion from HW1 cells treated with FC9402 (1μM) for 24, 48, and 72 hours). **(C)** Measurement of extracellular glutamine in media from HW1 cells treated with the SQOR inhibitor, FC9401 (1μM) for 24 hours. **(D)** Measurement of mitochondrial complexes I, II, IV, and V in HW1 cells treated with FC9402 (1μM) for 12, 24, 48, and 72 hours with tubulin used as a loading control. **(E-I)** Seahorse XFe mitostress test results for HW1 cells treated with the SQOR inhibitor, HTS07545 at increasing concentrations (1-10μM). *n*=5-7 replicates per group. **(E)** OCR of HTS07545 treated HW1 cells, **(F)** basal respiration, **(G)** maximum respiration, **(H)** ECAR, and **(I)** glycolysis determined from ECAR measured in cells treated with the SQOR inhibitor HTS07545. **(J)** ADP and ATP measurements in HW1 cells treated with vehicle control (VC) or 10μM HTS07545 (HTS). Results are represented as the ratio of ADP/ATP. *n*=6 replicates per group.

(K) Measurement of pyruvate secreted by HW1 cells treated with the SQOR inhibitor, HTS07545 (10μM) for 24 hours. **(L)** Lactate secretion from HW1 cells treated with HTS07545 (10μM) for 24, 48, and 72 hours). **(C)** Measurement of extracellular glutamine in media from HW1 cells treated with the SQOR inhibitor, HTS07545 (10μM) for 24 hours. Results are representative of 3 independent experiments **(A-M)**. Data are the mean ±s.d. **(A-C,F,G,I-M)**. *n*=4-7 replicates per group for each experiment. Statistics used: unpaired two-tailed *t*-test **(A,C,J,K,M)**, one-way ANOVA analysis with Tukey's multiple comparison test **(B,F,G,I,L)**, only comparisons with significant p-values <0.05 were indicated.

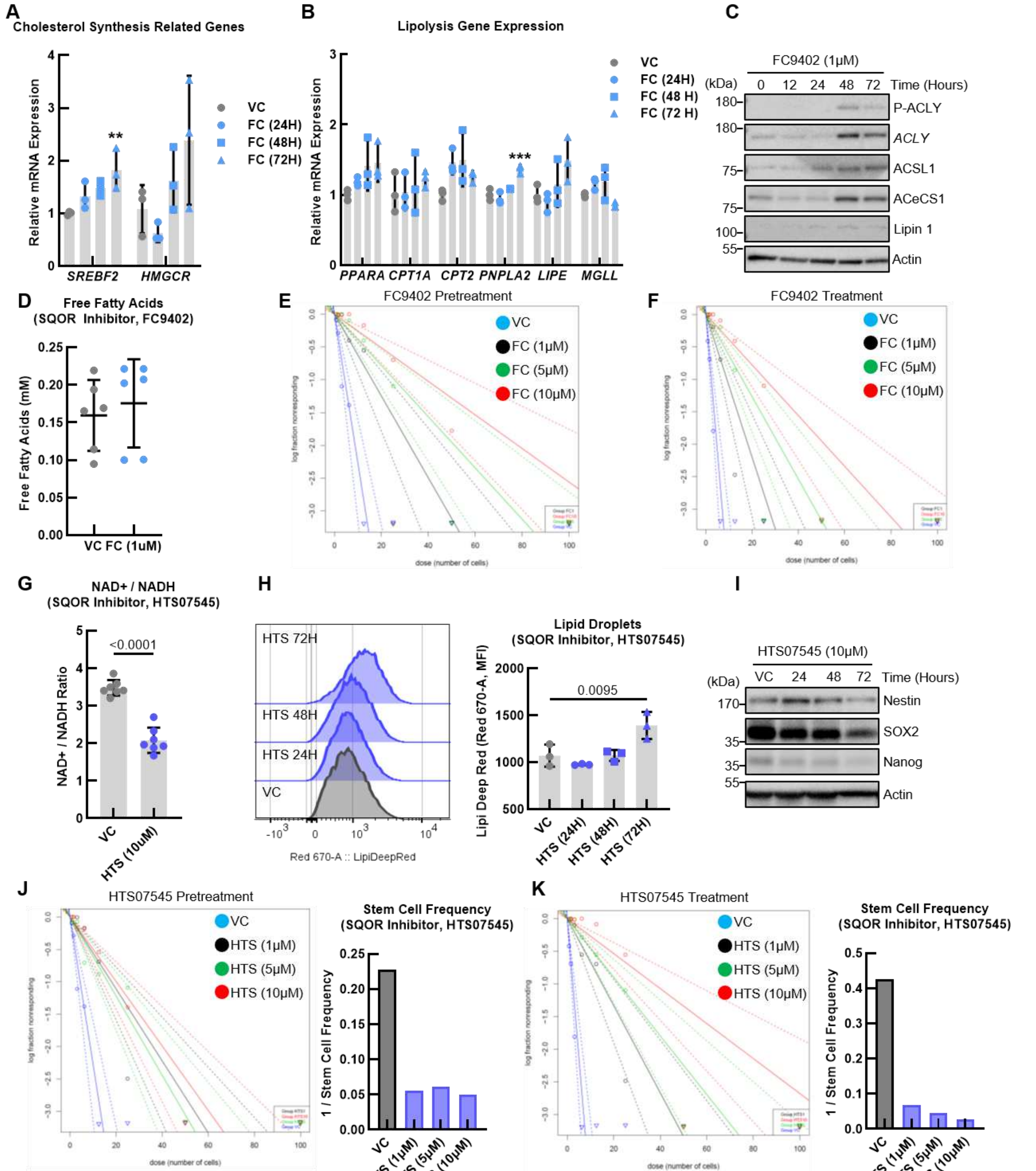

Group 1	Group 2	Chisq	DF	Pr(>Chisq)
HTS (1μM)	VC	52.6	1	4.03E-13
HTS (5μM)	VC	75.4	1	3.88E-18
HTS (10μM)	VC	107	1	3.73E-25

Group 1	Group 2	Chisq	DF	Pr(>Chisq)
HTS (1μM)	VC	28.2	1	1.11E-07
HTS (5μM)	VC	26	1	3.41E-07
HTS (10μM)	VC	33.3	1	7.89E-09

Supplementary Figure 5. Inhibition of sulfide oxidation in glioblastoma forces cells to accumulate lipids. (A) Evaluation of genes involved in lipolysis (PPARA, CPT1A, CPT2, PNPLA2, LIPE, and MGLL) by qRT-PCR in HW1 cells treated with 1μM FC9402 for 24, 48, and 72 hours. **(B)** Measurement of cholesterol synthesis related genes, (SREBF2 and

HMGCR) measured by qRT-PCR in HW1 cells treated for 24, 48, and 72 hours with 1μM FC9402. **(C)** Representative western blot analysis of enzymes involved in lipid synthesis. **(D)** Measurement of free fatty acids following inhibition of SQOR with FC9402 (1μM) in HW1 cells. **(E-F)** Extreme limiting dilution assay of L2 human glioblastoma cells pretreated or treated at the time of the assay with SQOR inhibitor, FC9402 (1-10μM). Results correspond with Figure 5J and 5K. **(G)** Measurement of NAD+ and NADH levels, in HW1 cells treated with the SQOR inhibitor, HTS07545 (10μM) for 24 hours. Results are represented as the ratio of NAD+/NADH. **(D)** Left, histogram of lipid droplets measured by flow cytometry using the fluorescent LipiDeep Red probe. Right, median fluorescence intensity (MFI) of LipiDeep Red signal measured in HW1 cells treated with the vehicle control or 10μM HTS07545 for increasing periods of time, 24, 48, and 72 hours. **(I)** Western blotting was performed to evaluate cancer stem cell markers Nestin, SOX2, and Nanog with tubulin used as a loading control in HTS07545 (10μM) treated HW1 cells. **(J-K)** Extreme limiting dilution assay of L2 human glioblastoma cells **(J)** pretreated or **(K)** treated at the time of the assay with SQOR inhibitor, HTS07545 (1-10μM). **(J)** Top left, ELDA results, top right results calculated and plotted as 1/stem cell frequency. Bottom, results of chi-square test. **(K)** Top left, ELDA results, top right results calculated and plotted as 1/stem cell frequency. Bottom, results of chi-square test. Results are representative of 3 independent experiments **(A-K)**. Data are the mean ±s.d. **(A,B,D,G,H)**. *n* indicates the number of replicates per group. Statistics used: one-way ANOVA analysis with Tukey's multiple comparison test **(C,D,H)**, chi-square test **(J,K)**, only comparisons with significant p-values <0.05 were indicated.

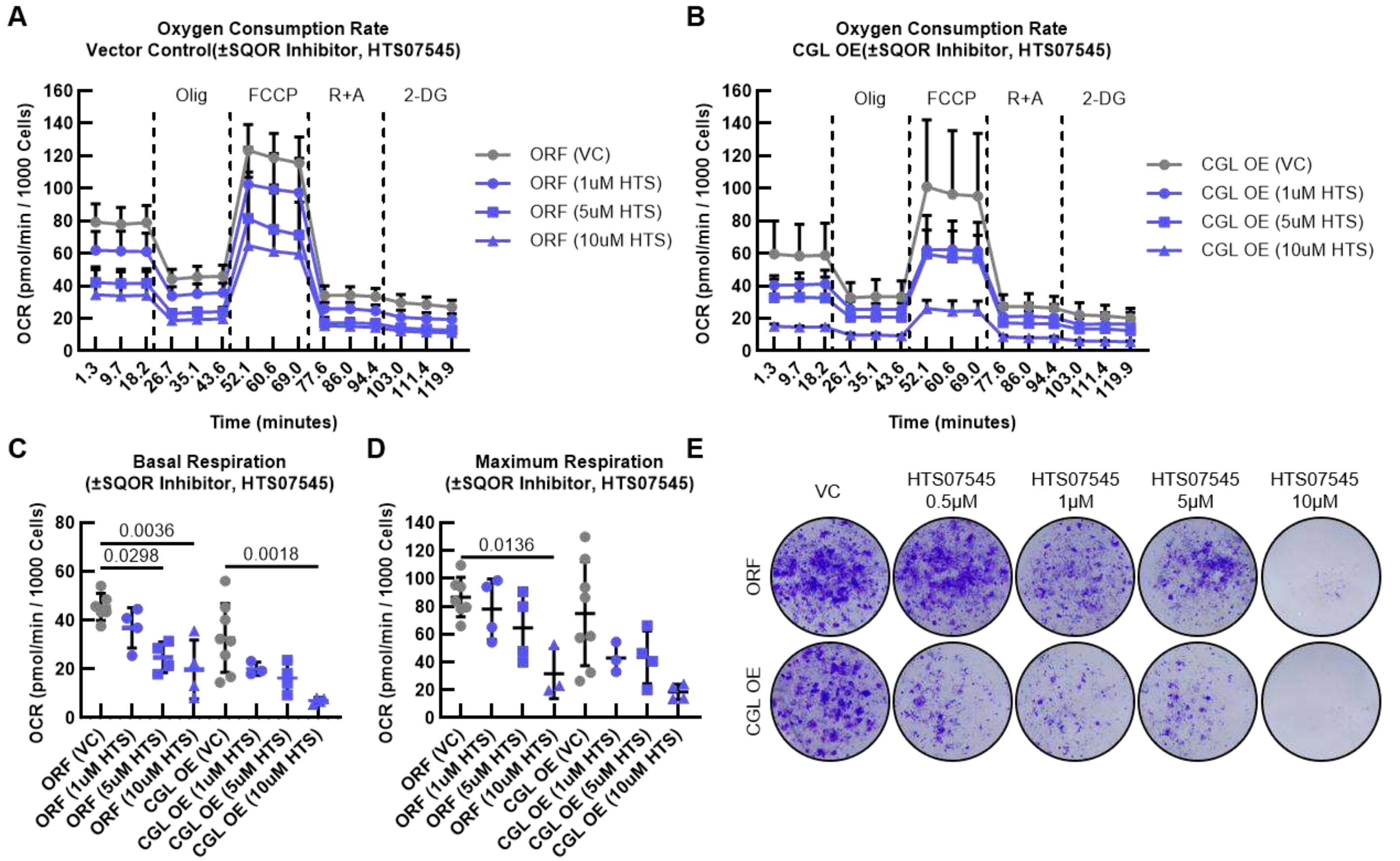

Supplementary Figure 6. SQOR inhibitor, HTS07545 shows increased effectiveness with increased H_2S production capacity. (A-D) Seahorse XFe mitostress test results *n*=3-7 replicates per group of open reading frame (ORF) controls and CGL overexpressing (CGL OE) HW1 cells. ORF and CGL OE cells were treated with either vehicle control (VC) or increasing concentrations of HTS07545 (1-10μM). Vehicle controls were the same as plotted in figure 6. **(A)** Oxygen consumption Rate (OCR) of ORF cells treated with HTS07545. **(B)** Oxygen consumption rate of CGL OE cells treated with HTS07545. **(C)** Basal respiration of ORF and CGL OE cells treated with HTS07545. **(D)** Maximum respiration of ORF and CGL OE cells treated with HTS07545. **(E)** Colony formation assay of ORF and CGL OE SB2b human glioblastoma cells treated with increasing concentrations (0.5-10μM) of HTS07545. **(A-E)**. Data are the mean ±s.d. **(C,D)**. *n*=3-7 replicates per group. Statistics used: one-way ANOVA analysis with Tukey's multiple comparison test **(C,D)**, only comparisons with significant p-values <0.05 were indicated.
